# Anisotropic lanthanide-based nano-clusters for imaging applications†
†Electronic supplementary information (ESI) available: Characterization details for clusters **1–3**. CCDC 1450317–1450319. For ESI and crystallographic data in CIF or other electronic format see DOI: 10.1039/c6fd00018e



**DOI:** 10.1039/c6fd00018e

**Published:** 2016-03-31

**Authors:** Xiaoping Yang, Shiqing Wang, Tyler L. King, Christopher J. Kerr, Clement Blanchet, Dmitri Svergun, Robert Pal, Andrew Beeby, Jamuna Vadivelu, Katherine A. Brown, Richard A. Jones, Lijie Zhang, Shaoming Huang

**Affiliations:** a College of Chemistry and Materials Engineering , Wenzhou University , Zhejiang Key Laboratory of Carbon Materials , Wenzhou 325035 , China . Email: smhuang@wzu.edu.cn ; Tel: +86 577 88373064; b The University of Texas at Austin , Department of Chemistry , Austin , Texas 78712 , USA . Email: rajones@cm.utexas.edu ; Tel: +1 512 4711706; c European Molecular Biology Laboratory , Hamburg Unit , EMBL c/o DESY , Hamburg , 22607 , Germany; d Department of Chemistry , University of Durham , South Road , Durham , DH1 3LE , UK; e Department of Medical Microbiology , University of Malaya , Kuala Lumpur 50603 , Malaysia; f Cavendish Laboratory , Department of Physics , University of Cambridge , Cambridge CB3 0HE , UK . Email: kb518@cam.ac.uk

## Abstract

We have developed a new class of lanthanide nano-clusters that self-assemble using flexible Schiff base ligands. Cd–Ln and Ni–Ln clusters, [Ln_8_Cd_24_(L^1^)_12_(OAc)_39_Cl_7_(OH)_2_] (Ln = Nd, Eu), [Eu_8_Cd_24_(L^1^)_12_(OAc)_44_], [Ln_8_Cd_24_(L^2^)_12_(OAc)_44_] (Ln = Nd, Yb, Sm) and [Nd_2_Ni_4_(L^3^)_2_(acac)_6_(NO_3_)_2_(OH)_2_], were constructed using different types of flexible Schiff base ligands. These molecular nano-clusters exhibit anisotropic architectures that differ considerably depending upon the presence of Cd (nano-drum) or Ni (square-like nano-cluster). Structural characterization of the self-assembled particles has been undertaken using crystallography, transmission electron microscopy and small-angle X-ray scattering. Comparison of the metric dimensions of the nano-drums shows a consistency of size using these techniques, suggesting that these molecules may share similar structural features in both solid and solution states. Photophysical properties were studied by excitation of the ligand-centered absorption bands in the solid state and in solution, and using confocal microscopy of microspheres loaded with the compounds. The emissive properties of these compounds vary depending upon the combination of lanthanide and Cd or Ni present in these clusters. The results provide new insights into the construction of novel high-nuclearity nano-clusters and offer a promising foundation for the development of new functional nanomaterials.

## Introduction

Lanthanide nanomaterials are being increasingly investigated for applications in bio-imaging and molecular recognition,^1^ due in part to their advantageous photophysical properties, which include a very large pseudo-Stokes shift between the excitation and emission wavelengths, the absence of photo-bleaching, long lived excited states and narrow emission bands.^1,2^ Since emissions *via* 4f–4f transitions are parity forbidden,^3^ Ln^3+^ ions usually have a relatively long photoluminescence lifetime of the excited state. This can allow for gated and time-resolved photoluminescence detection and thus can eliminate interference from short-lived autofluorescence.

High-nuclearity heterometallic nano-clusters composed of d-block transition metals and lanthanide ions (Ln) have received extensive attention due not only to their aesthetically stunning molecular structures but also their versatile applications in optoelectronics, magnetism, and as probes in biological systems.^4^ Most trivalent lanthanide ions exhibit long-lived (in the microsecond-to-millisecond range) and line-like emission bands at characteristic wavelengths, *e.g.* Tb^3+^ (green), Eu^3+^ (red) and Yb^3+^, Nd^3+^, and Er^3+^ (near infra-red, NIR). These characteristics allow time-gated detection. More specifically, the NIR emitters have some potential applications in bioassays and luminescent probes because of the transparency of biological tissues to electromagnetic radiation in the range of 0.8–1.3 μm.^5^ In high-nuclearity d–f clusters, light-absorbing d-block metal chromophores (*i.e.* Pt^II^,^6a,b^ Ru^II^,^6c,d^ Zn^II^,^6e,f^ and Cd^II^ ^6g,h^) can act as sensitizers for luminescence from Ln(iii) centers following d → f energy-transfer. Many reports have so far been focused on high-nuclearity 3d–4f clusters, such as Cu/Ln,^7*a*^ Mn/Ln^7b,c^ and Ni/Ln^7d,e^ cluster systems, in order to study their magnetic properties as single-molecule magnets. In contrast, high-nuclearity 4d–f systems with luminescence properties have received much less attention.

**Scheme 1:**
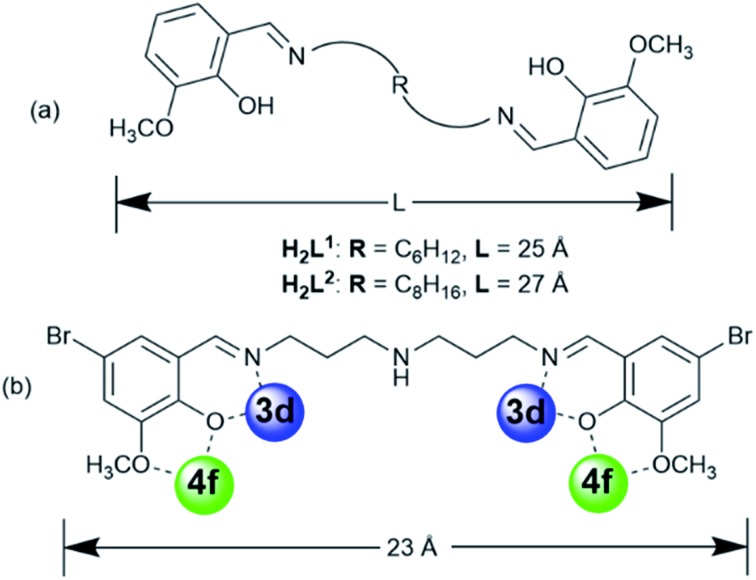
(a) Flexible Schiff base ligands; (b) the “stretching” coordination mode of H_2_L^3^.

Compartmental Schiff bases with two dissimilar metal-binding sites, one being specific for the d metal ion and another for the f metal ion, have been employed to synthesize d–f heteronuclear clusters.^8^ Recent studies in our laboratories have focused on the construction of luminescent d–f clusters with flexible Schiff base ligands.^9^ Flexible ligands may provide more possibilities for the construction of unique frameworks because of their freedom of conformation. In our previous studies, the use of Schiff base ligands which have flexible carbon–carbon backbones containing from 2 to 4 methylene (CH_2_) units, resulted in a variety of binuclear,^10*a*^ trinuclear,^10*b*^ tetranuclear,^10*c*^ and hexanuclear^10d,e^ 3d–4f complexes (3d = Zn, Cu and Ni). Recently, we discovered a series of 24-metal Cd–Ln complexes [Ln_6_Cd_18_(L^1^)_9_Cl_8_(OAc)_28_] from reactions of a flexible Schiff base ligand, *N*,*N*′-bis(5-bromo-3-methoxysalicylidene)hexane-1,6-diamine (H_2_L^1^), which has a 6 carbon backbone (Scheme 1), with Cd(OAc)_2_·4H_2_O and LnCl_3_·6H_2_O.^9*b*^ The structures of these metal clusters are often influenced by a variety of factors such as the ligand structure, the nature of the counter anions, and the pH value of the surrounding environment. As part of our continuing studies focused on the construction of luminescent polynuclear lanthanide-based frameworks, we report here seven d–f clusters with H_2_L^1^, H_2_L^2^ and long-chain Schiff base ligand *N*,*N*′-bis(5-bromo-3-methoxysalicylidene) (*N*′′-(3-amion-propyl)-propane)-1,6-diamine (H_2_L^3^), which has a flexible (CH_2_)_3_NH(CH_2_)_3_ backbone (Scheme 1b). They are [Ln_8_Cd_24_(L^1^)_12_(OAc)_39_Cl_7_(OH)_2_] (Ln = Nd (**1**) and Eu (**2**)), [Nd_2_Ni_4_(L^3^)_2_(acac)_6_(NO_3_)_2_(OH)_2_] (**3**), [Ln_8_Cd_24_(L^2^)_12_(OAc)_48_] (Ln = Eu (**4**) and Sm (**5**)) and [Ln_8_Cd_24_(L^2^)_12_(OAc)_44_Cl_4_] (Ln = Nd (**6**) and Yb (**7**)). **1** and **2** were synthesized from the reactions of H_2_L^1^ with CdCl_2_·2H_2_O and Ln(OAc)_3_·4H_2_O in the presence of NaOH. Interestingly, differing from our previously reported Cd–Ln complexes [Ln_6_Cd_18_(L^1^)_9_Cl_8_(OAc)_28_], **1** and **2** exhibit 32-metal anisotropic nano-drum-like architectures. Hydroxide (OH^–^) anions are found in the structures of **1** and **2**, indicating that the basic environment favors the formation of these complexes. The structures appear to be ligand and anion dependent. Compared with H_2_L^1^ and H_2_L^2^, H_2_L^3^ features a backbone NH group. Thus, **3** shows a 6-metal square-like structure. The flexible Schiff-base ligands H_2_L^1^, H_2_L^2^ and H_2_L^3^ exhibit “stretched” coordination modes with metal ions (Scheme 1b), resulting in the large sizes of **1–7**. For example, the sizes of **1** and **2** are approximately 19 × 20 × 20 Å, which are much larger than most other d–f Schiff base polynuclear complexes thus far reported. To further explore the self-assembly properties and imaging of these molecules, we also report preliminary small angle X-ray scattering measurements and confocal imaging of Cd–Ln complexes containing the H_2_L^2^ Schiff base ligand.

## Experimental section

### General information

All reactions were performed under dry oxygen-free dinitrogen atmospheres using standard Schlenk techniques. The Schiff-base ligands H_2_L^1,2,3^ were prepared according to well-established procedures.^11^ Physical measurements: NMR: VARIAN UNITY-plus 600 spectrometer (^1^H, 600 MHz) at 298 K; IR: Nicolet IR 200 FTIR spectrometer. Elemental analyses (C, H, N) were carried out on a EA1112 elemental analyser. Melting points were obtained in sealed glass capillaries under dinitrogen and are uncorrected. Conductivity measurements were carried out with a DDS-11 conductivity bridge for 10^–3^ M solutions in CH_3_CN. Transmission electron microscopy (TEM) images were recorded on a JEOL JEM-1200EX transmission electron microscope. Scanning electron microscopy (SEM) images were recorded on a Nova NanoSEM 200 scanning electron microscope. Absorption spectra were obtained on a UV-3600 spectrophotometer, and excitation and emission spectra on a QuantaMaster PTI fluorimeter.

### Syntheses

#### [Nd_8_Cd_24_(L^1^)_12_(OAc)_39_Cl_7_(OH)_2_] (**1**)

CdCl_2_·2H_2_O (0.0439 g, 0.20 mmol), Nd(OAc)_3_·4H_2_O (0.0393 g, 0.10 mmol) and H_2_L^1^ (0.0770 g, 0.20 mmol) were dissolved in MeOH (20 mL) at room temperature, and a solution of NaOH in EtOH (0.04 mol L^–1^, 10 mL) was then added. The resulting solution was stirred and heated under reflux (30 min). The mixture was allowed to cool and was then filtered. Diethyl ether was allowed to diffuse slowly into the filtrate at room temperature and pale yellow crystals were obtained after one week. The crystals were filtered off, washed with EtOH (5 mL) and dried in the air. Yield (based on CdCl_2_·2H_2_O): 0.0623 g (63%). Mp > 217 °C (dec.). Found: C, 36.55; H, 4.71; N, 2.39%. Calc. for C_342_H_428_Cd_24_Cl_7_Nd_8_N_24_O_128_(EtOH)_6_(EtOEt)_2_(MeOH)_8_(H_2_O)_15_: C, 37.08; H, 4.56; N, 2.81%. IR (CH_3_OH, cm^–1^): 3091 (w), 2935 (w), 1630 (s), 1575 (m), 1472 (m), 1350 (w), 1309 (m), 1239 (m), 1213 (s), 1082 (m), 1045 (m), 962 (w), 847 (m), 795 (s), 672 (w), 616 (w).

#### [Eu_8_Cd_24_(L^1^)_12_(OAc)_39_Cl_7_(OH)_2_] (**2**)

The procedure was the same as that for **1** using Eu(OAc)_3_·4H_2_O (0.0418 g, 0.10 mmol). Pale yellow single crystals of **2** were formed after one week. Yield (based on CdCl_2_·2H_2_O): 0.0519 g (52%). Mp > 220 °C (dec.). Found: C, 36.72; H, 4.57; N, 2.51%. Calc. for C_342_H_428_Cd_24_Cl_7_Eu_8_N_24_O_128_(EtOH)_5_(EtOEt)_2_(MeOH)_6_(H_2_O)_12_: C, 37.00; H, 4.43; N, 2.83%. IR (CH_3_OH, cm^–1^): 3099 (w), 1637 (m), 1578 (s), 1470 (s), 1410 (m), 1341 (m), 1318 (w), 1210 (s), 1088 (m), 1039 (m), 962 (m), 855 (m), 798 (s), 673 (m), 638 (m), 615 (w).

#### [Nd_2_Ni_4_(L^3^)_2_(acac)_6_(NO_3_)_2_(OH)_2_] (**3**)

The procedure was the same as that for **1** using Ni(acac)_2_·2H_2_O (0.1534 g, 0.52 mmol), Nd(NO_3_)_3_·6H_2_O (0.0526 g, 0.12 mmol) and H_2_L^3^ (0.26 mmol, 0.1448 g). Pale green single crystals of **3** were formed after two weeks. Yield (based on Nd(NO_3_)_3_·6H_2_O): 0.0756 g (52%). Mp > 178 °C (dec.). Found: C, 26.39; H, 2.13; N, 4.16%. Calc. for C_56_H_50_Br_4_Nd_2_N_8_Ni_4_O_52_: C, 26.86; H, 2.00; N, 4.48%. IR (CH_3_OH, cm^–1^): 2933 (m), 1631 (m), 1575 (s), 1432 (s), 1400 (s), 1301 (m), 1205 (s), 1072 (m), 1022 (w), 970 (w), 845 (w), 730 (s), 666 (m).

#### [Eu_8_Cd_24_(L^2^)_12_(OAc)_48_] (**4**)

Cd(OAc)_2_·2H_2_O (0.1382 g, 0.52 mmol), Eu(OAc)_3_·4H_2_O (0.0502 g, 0.12 mmol) and H_2_L^2^ (0.1073 g, 0.26 mmol) were dissolved in MeOH (30 mL) at room temperature and 4 drops of Et_3_N were added. The resulting solution was stirred and heated under reflux for 1 hour. The mixture was then allowed to cool to room temperature and was gravity filtered. Diethyl ether was allowed to diffuse slowly into the filtrate at room temperature and yellow crystals were obtained after one week. The crystals were washed with EtOH (5 mL) and Et_2_O (5 mL) and collected and dried in the air. Yield (based on Eu(OAc)_3_·4H_2_O): 0.0478 g (53%). Mp > 216 °C (dec.). IR (neat, cm^–1^) 2926 (w), 2825 (w), 1630 (m), 1552 (s), 1403 (s), 1345 (m), 1305 (m), 1210 (s), 1167 (m), 1097 (m), 1078 (m), 1017 (w), 964 (w), 852 (w), 735 (s), 666 (m).

#### [Sm_8_Cd_24_(L^2^)_12_(OAc)_48_] (**5**)

The synthesis of **5** was the same as that for **4** using Sm(OAc)_3_·4H_2_O (0.0482 g, 0.12 mmol). Yellow crystals of **5** were formed after one week. Yield (based on Sm(OAc)_3_·4H_2_O): 0.0419 g (47%). Mp > 207 °C (dec.). IR (CH_3_OH, cm^–1^): 2926 (w), 1631 (m), 1575 (s), 1558 (s), 1471 (m), 1410 (s), 1306 (m), 1212 (s), 1074 (m), 1016 (w), 966 (w), 854 (w), 734 (s), 665 (w).

#### [Nd_8_Cd_24_(L^2^)_12_(OAc)_44_Cl_4_] (**6**)

The synthesis of **6** was the same as that for **4** using NdCl_3_·6H_2_O (0.0431 g, 0.12 mmol). Yellow crystals were obtained after one week. Yield (based on NdCl_3_·6H_2_O): 0.0712 g (45%). Mp > 209 °C (dec.). IR (CH_3_OH, cm^–1^): 3374 (w), 2930 (w), 1631 (m), 1572 (s), 1470 (m), 1410 (s), 1347 (w), 1307 (m), 1238 (m), 1212 (s), 1080 (m), 1048 (m), 963 (w), 849 (m), 738 (s), 673 (s), 640 (m).

#### [Yb_8_Cd_24_(L^2^)_12_(OAc)_44_Cl_4_] (**7**)

The synthesis of **7** was the same as that for **4** using YbCl_3_·6H_2_O (0.0466 g, 0.12 mmol). Pale yellow single crystals of **7** were formed after one week. Yield (based on YbCl_3_·6H_2_O): 0.0612 g (37%). Mp > 209 °C (dec.). IR (CH_3_OH, cm^–1^): 2930 (w), 1635 (m), 1576 (s), 1468 (m), 1407 (s), 1340 (w), 1309 (m), 1238 (m), 1212 (s), 1078 (m), 1020 (m), 962 (m), 854 (m), 735 (s), 676 (m), 638 (m).

### X-ray analysis

Data were collected using a Smart APEX CCD diffractometer with graphite monochromated Mo-Kα radiation (*λ* = 0.71073 Å) at 223 K. The data set was corrected for absorption based on multiple scans and reduced using standard methods. Data reduction was performed using DENZO-SMN.^12^ The structures were solved by direct methods and refined anisotropically using full-matrix least-squares methods with the SHELX 97 program package.^12^ Coordinates of the non-hydrogen atoms were refined anisotropically, while hydrogen atoms were included in the calculation isotropically but not refined. Neutral atom scattering factors were taken from Cromer and Waber.^14^

For the crystal structures of **1** and **2**, some uncoordinated solvent molecules such as CH_3_OH, C_2_H_5_OH, C_2_H_5_OC_2_H_5_ and H_2_O molecules were found to be badly disordered. Attempts to model the disorder were unsatisfactory. The contributions to the scattering factors due to these solvent molecules were removed by use of the utility SQUEEZE^15*a*^ in PLATON98.^15b,c^ PLATON98 was used as incorporated in WinGX.^15*d*^ Crystallographic data for **1–3** (CCDC reference numbers ; 1450317–1450319) are presented in Table S1 and selected bond lengths are given in Tables S2–S4.†

**1**: C_342_H_428_Cd_24_Cl_7_N_24_Nd_8_O_128_, orthorhombic, space group *Pna*2(1), *a* = 54.734(11), *b* = 27.619(12), *c* = 35.243(18) Å, *α* = 90°, *β* = 90°, *γ* = 90°, *V* = 53 277(18) Å^3^, *Z* = 4, Dc = 1.374 g cm^–3^, *μ*(Mo-Kα) = 1.798 mm^–1^, *F*(000) = 21 692, *T* = 223 K. *R*_1_ = 0.1018, w*R*_2_ = 0.2608 for 90 178 independent reflections with a goodness-of-fit of 1.038.

**2**: C_342_H_428_Cd_24_Cl_7_N_24_Eu_8_O_128_, orthorhombic, space group *Pna*2(1), *a* = 54.106(11), *b* = 27.127(5), *c* = 34.876(7) Å, *α* = 90°, *β* = 90°, *γ* = 90°, *V* = 51 189(18) Å^3^, *Z* = 4, Dc = 1.438 g cm^–3^, *μ*(Mo-Kα) = 2.040 mm^–1^, *F*(000) = 21 788, *T* = 223 K. *R*_1_ = 0.1035, w*R*_2_ = 0.2090 for 86 518 independent reflections with a goodness-of-fit of 0.922.

**3**: C_56_H_50_Br_4_N_8_Nd_2_Ni_4_O_52_, monoclinic, space group *P*2_1_/*c*, *a* = 15.462(3), *b* = 19.156(4), *c* = 19.871(4) Å, *α* = 90°, *β* = 107.63°(3), *γ* = 90°, *V* = 5609.2(19) Å^3^, *Z* = 2, Dc = 1.481 g cm^–3^, *μ*(Mo-Kα) = 3.073 mm^–1^, *F*(000) = 2452, *T* = 223 K. *R*_1_ = 0.0781, w*R*_2_ = 0.2092 for 9806 independent reflections with a goodness-of-fit of 1.061.

### Small-angle X-ray scattering (SAXS)

SAXS data for [Eu_8_Cd_24_(L^2^)_12_(OAc)_48_] (**4**) were collected on the SAXS beamline P12 at the PETRA III storage ring (Deutsches Elektronen-Synchrotron, Hamburg).^13^ Using a PILATUS 2M pixel detector at a sample-detector distance of 3.0 m and at an energy of 9.7 keV (*λ* = 1.28 Å), the range of momentum transfer 0.01 < *s* < 0.45 Å^–1^ was covered (*s* = 4π sin *θ*/*λ*, where 2*θ* is the scattering angle). Solute concentrations of 1.8 and 3.7 mg mL^–1^ were measured in MeOH/MeCN (50 : 50) at 20 °C. Samples were loaded manually into the observation capillary. Primary data processing steps were performed using the automated data pipeline SASFLOW.^16^

### Polystyrene bead loading

Luminex MicroPlex microspheres (6 μm cross-linked polystyrene beads with surface carboxyl groups) were prepared for loading by drying 12.5 × 10^6^ beads under high vacuum for 24 hours. The dried beads were resuspended in a chloroform/MeOH solution (1 mL, 50 : 50) containing 5 mg of sample. This suspension was rotated slowly for 2 days. The suspension was then centrifuged, the supernatant discarded and the beads washed with MeOH (3 × 1 mL) followed by drying under high vacuum (24 h).

### Confocal microscopy of polystyrene beads

Images were recorded using a Leica SP5 II confocal microscope equipped with a HCX PL APO 63x/1.40 NA LambdaBlue Oil immersion objective. Data were collected using 5× digital magnification at a 400 Hz/line scan speed (4 line average, bidirectional scanning) at 355 nm (3^rd^ harmonic NdYAG laser) with 3 mW laser power. In order to achieve excitation with maximal probe emission, the microscope was equipped with a triple channel imaging detector, comprising two conventional PMT systems and a HyD hybrid avalanche photodiode detector. The latter part of the detection system, when operated in the BrightRed mode, is capable of improving imaging sensitivity by 25%, reducing signal to noise by a factor of 5. Frame size was determined at 2048 × 2048 pixels, with a 0.6 airy disc unit determining the applied pinhole diameter rendering one voxel to correspond to 24.02 × 24.02 nm (frame size 49.16 × 49.16 μm) with a section thickness of 380 nm. This particular LSCM is equipped with a novel structural illumination module called PhMoNa (achievable resolution 62 × 62 × 280 nm).^17^ To detect sensitized Ln emission, a corresponding detection window of 400–800 nm was used to record images using only the above detailed 355 nm laser line.

## Results and discussion

**Fig. 1:**
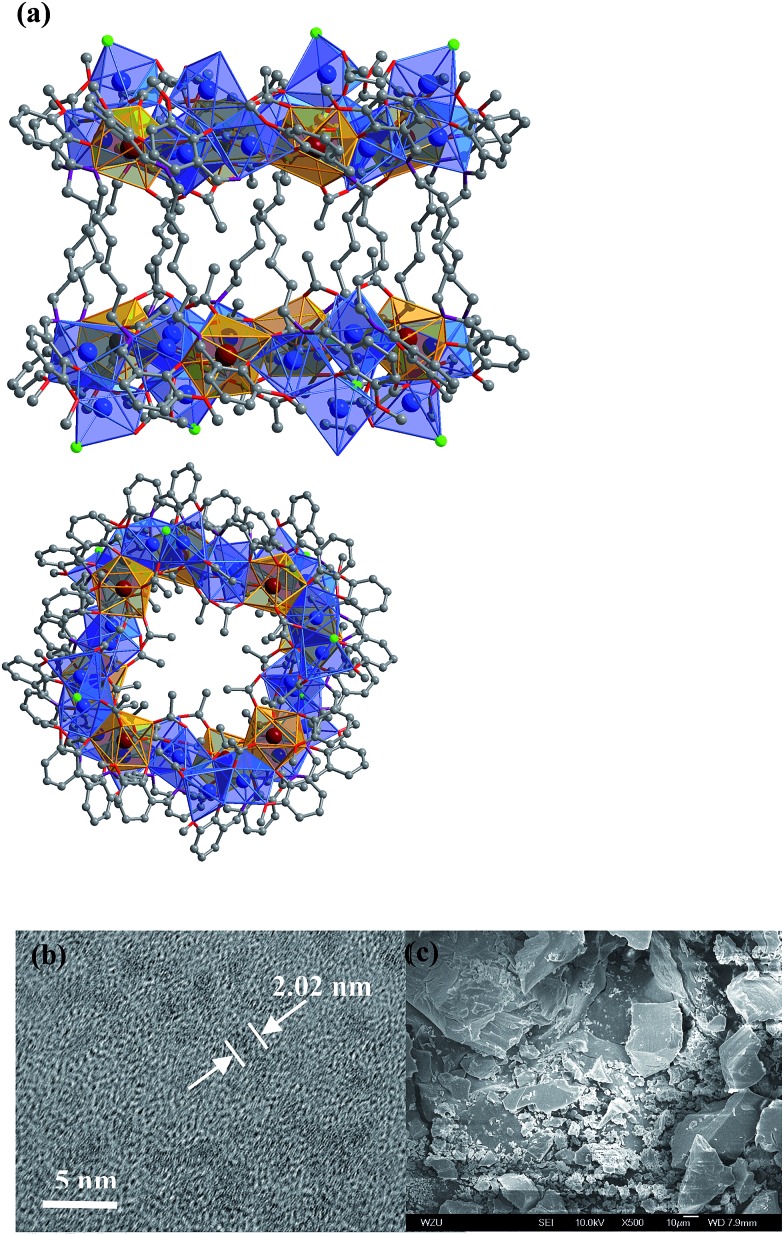
(a) Two views of the nano-drum-like structure of **1**: viewed along the *b*-axis (top) and the *ac*-axis (lower) (Nd^3+^: brown; Cd^2+^: blue; Cl^–^: green); (b and c) TEM and SEM images of **1**.

In the presence of NaOH (0.01 mol L^–1^), reactions of H_2_L^1^ with CdCl_2_·2H_2_O and Ln(OAc)_3_·4H_2_O in refluxing methanol/ethanol produced yellow solutions from which **1** and **2** were isolated as pale yellow crystalline solids. **1** and **2** are isomorphous and have 32-metal drum-like structures. Two views of the crystal structure of **1** are shown in Fig. 1a. The top view is essentially a side-on view while the lower one is looking down into the top of the drum. The ends of the drum are created by two rings of 16 metals (4 Nd(iii) and 12 Cd(ii)) coordinated to half of the N, O binding groups of the 12 Schiff base ligands. The sides of the drum are formed by the –(CH_2_)_6_– linkers of the Schiff base ligands. Regarding the two Nd_4_Cd_12_ rings, one includes four Cl^–^, one OH^–^ and nineteen OAc^–^ anions and the other has three Cl^–^, one OH^–^ and twenty OAc^–^ anions to balance the charge of the cluster. In **1**, the Cd–O and Cd–N bond lengths range from 2.163 Å to 2.646 Å and 2.210 Å to 2.406 Å, respectively. The Nd–O and Nd–N bond lengths range from 2.208 Å to 2.652 Å and 2.527 Å to 2.650 Å, respectively. In **1** and **2**, each Ln^3+^ ion and its closest two Cd^2+^ ions are linked by phenolic oxygen atoms of the L^1^ ligand and OAc^–^ anions.

**Fig. 2:**
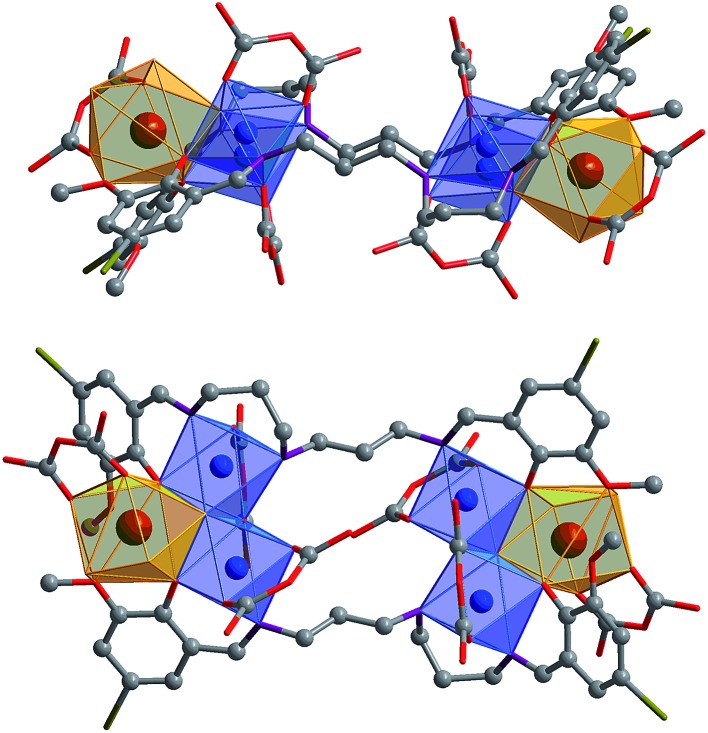
Two views of the square-like structure of **3**: viewed along the *ac*-axis (top) and the *b*-axis (lower) (Nd^3+^: brown; Ni^2+^: blue).

The Cd(ii) ion tends to have relatively high coordination numbers (6–8) and exhibits variable coordination geometries, somewhat similar to Ln(iii). This geometry may favor the formation of high-nuclearity Cd–Ln clusters **1** and **2**. Compared with Cd(ii), the Ni(ii) ion tends to have lower coordination numbers (4–6) and exhibits some common coordination geometries such as square-planar, tetragonal pyramid and octahedral. Thus, the reaction of H_2_L^3^ with Ni(acac)_2_·2H_2_O and Nd(NO_3_)_3_·6H_2_O under the same experimental conditions as above produced green solutions from which **3** was isolated as a green crystalline solid. Two views of the square-like structure of **3** are shown in Fig. 2. The top view is looking down into the top of the rectangle while the lower view is essentially a side-on view. The X-ray structure of **3** reveals a centrosymmetric core with two equivalent NdNi_2_(acac)_3_(NO_3_)(OH) moieties linked by two L^2^ ligands. In each NdNi_2_(acac)_3_(NO_3_)(OH) moiety, the Nd^3+^ ion is 8-coordinate, surrounded by eight oxygen atoms from two L^2^ ligands, one acac^–^, one NO_3_^–^ and one OH^–^ ion. Both Ni^2+^ ions have bi-pyramidal geometries. They are bridged by one acac^–^ and one OH^–^ ion with a separation of 3.237 Å. The Nd^3+^ ion and two Ni^2+^ ions are linked by phenolic oxygen atoms of the L^2^ ligand and OH^–^ anions. The average distance between the Nd^3+^ and Ni^2+^ ions is 3.413 Å.

It is noticeable that the Schiff base ligands exhibit a “stretched” coordination mode with metal ions in **1–3** (Scheme 1b). This may be due to the fact that, compared with those Schiff base ligands with shorter carbon-carbon backbones, there is less of a chelating or templating effect.^1,2^ Thus the heights of the drum-like and square-like structures are mainly decided by the lengths of the Schiff base ligands, resulting in the formation of nanoscale clusters. The molecular dimensions of **1** and **2** are bigger than those of **3** (*i.e.* 19 × 20 × 20 Å for **1** *vs.* 8 × 12 × 18 Å for **3**). It is also possible to obtain images of these Cd–Ln molecular nanoparticles using transmission electron microscopy (TEM). Dilute solutions of **1** in CH_3_CN were contacted with a Cu grid and the solvent was carefully evaporated under vacuum. The TEM images obtained (Fig. 1b) show uniform nanoparticles with diameters measuring approximately 2.02 nm which corresponds well with the diameter of the 16-metal ring end of the drum found in the crystal structure, indicating that the nano-cluster retains its unique molecular structure in solution. In Fig. 1c a panoramic scanning electron microscopy (SEM) image shows the crystalline nature of **1**.

The nature of **1** in solution has been probed using molar conductivity and ^1^H NMR studies. Molar conductivity studies of **1** in CH_3_CN also confirm that this molecular nanoparticle is neutral in solution, in accordance with the solid state structure. The ^1^H NMR spectrum of **1** in CDCl_3_ contains multiple broad peaks ranging from –17 to +18 ppm, due to the paramagnetic Nd^3+^ ion. The ^1^H NMR spectrum of **1** remains unchanged for several weeks, indicating that it is stable in solution (Fig. S1†).

**Fig. 3:**
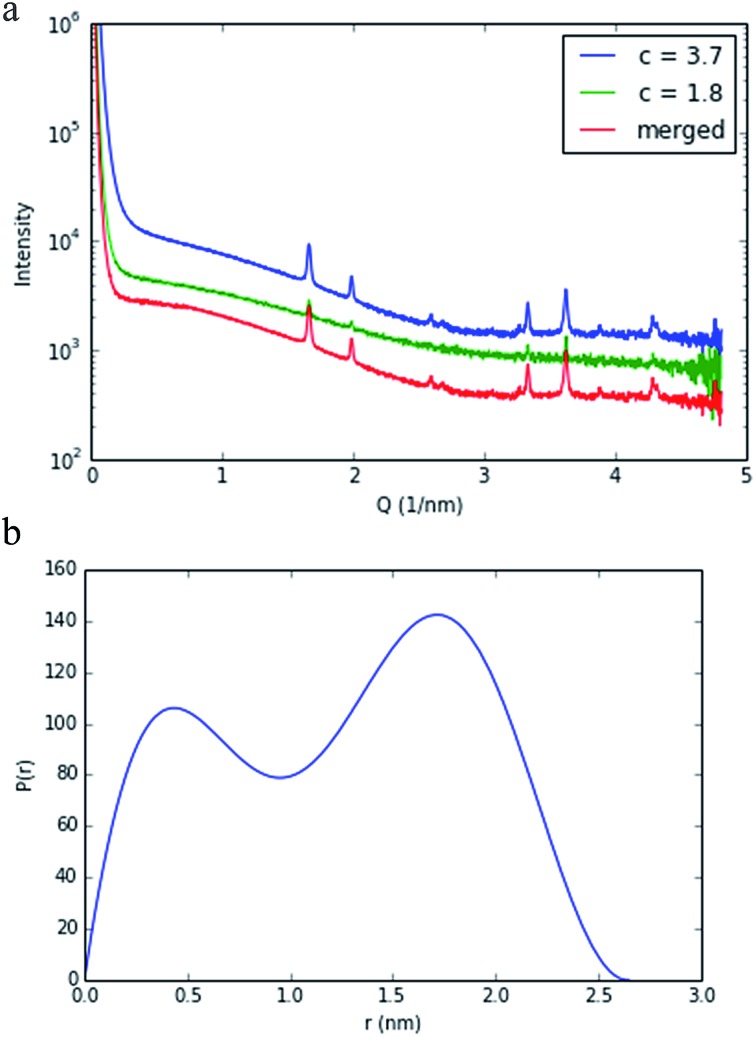
SAXS analysis of compound **4**. (a) SAXS curves of data collected at 1.8 and 3.7 mg mL^–1^ are shown with the merged curve that was used as an input to derive (b) the particle distance function, *P*(*r*), plot.

The nature of **4** in solution has been probed using small-angle X-ray scattering (SAXS). SAXS data were obtained at 1.8 and 3.7 mg mL^–1^, as described above, and merged using the ALMERGE^18^ software as shown in Fig. 3a. Bragg peaks in the scattering data appear to be arising from undissolved material still in a crystalline state. Evaluation of the particle distance distribution function *P*(*r*) was undertaken using GNOM^19^ and shows a bi-modal distribution, with a maximum particle dimension of approximately 2.7 nm, consistent with the crystal structure of this nano-drum.^20^ However, further studies are required to establish conditions that are purely monodisperse, without the presence of undissolved crystalline material and/or nano-cluster substructures.

**Fig. 4:**
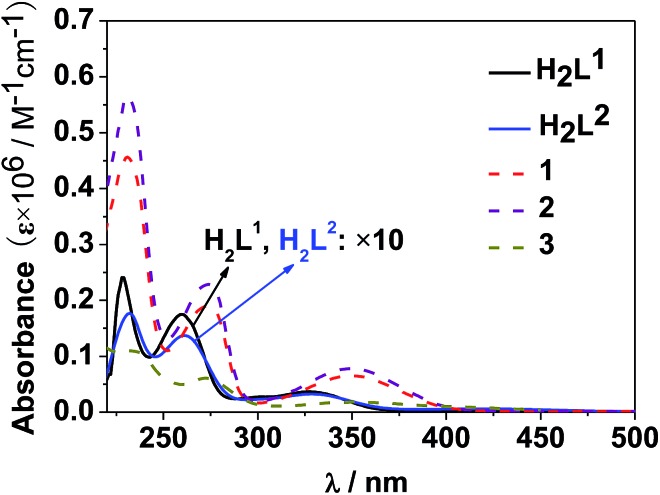
UV-vis spectra of the free ligands H_2_L^1,2^ and clusters **1–3** in CH_3_CN (*C* = 10^–8^ to 10^–7^ M).

**Fig. 5:**
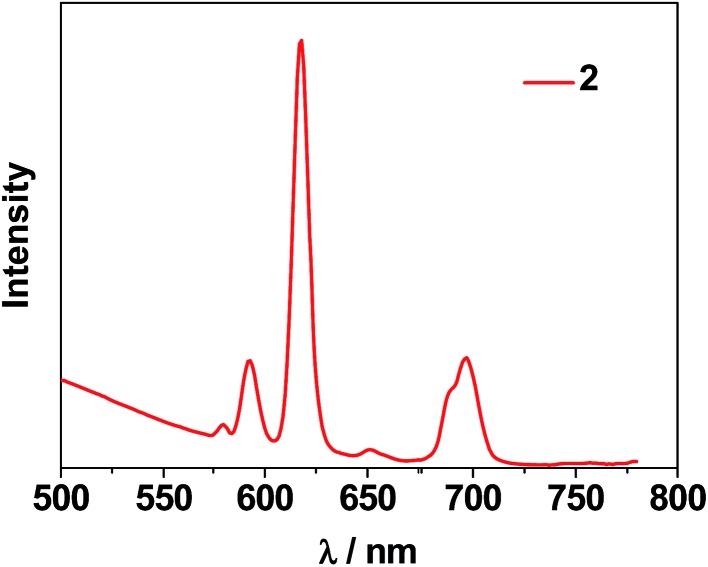
The visible emission spectrum of **2** in CH_3_CN.

**Fig. 6:**
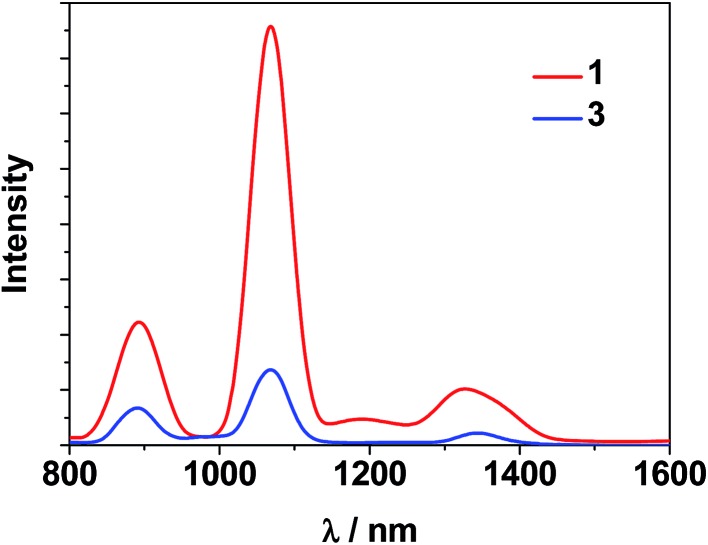
NIR emission spectra of clusters **1** and **3** with the same absorbance value at *λ*_ex_ = 355 nm in CH_3_CN.

The photophysical properties of **1–3** were studied in both solution and the solid state. The d–f clusters' absorption bands in the UV-vis region are all red-shifted compared to those of the ligands alone (H_2_L^1,2^) (Fig. 4). For the free ligands, H_2_L^1,2^, excitations produce broad emission bands at *λ*_max_ = 515 nm and 509 nm, respectively (Fig. S2†). The emission and excitation spectra of **1–3** in CH_3_CN are shown in Fig. 5, 6 and S3.† Upon excitation of the ligand-centered absorption bands, **1** and **3** show the NIR luminescence of Nd^3+^ (^4^F_3/2_ → ^4^I_*j*/2_ transitions, *j* = 9, 11 and 13). As shown in Fig. 6, the emissions at 873 nm can be assigned to the ^4^F_3/2_ → ^4^I_9/2_ transition, 1063 nm to the ^4^F_3/2_ → ^4^I_11/2_ transition and 1350 nm to the ^4^F_3/2_ → ^4^I_13/2_ transition of Nd^3+^. The free Schiff base ligands H_2_L^1,2^ and Nd(OAc)_3_·4H_2_O do not exhibit NIR luminescence in CH_3_CN under similar conditions. Upon excitation of the ligand centered absorption bands, **2** shows typical visible emission bands of the Eu^3+^ ion (^5^D_0_ → ^7^F_*j*_ transitions, *j* = 0, 1, 2, 3 and 4). As shown in Fig. 5, the appearance of the symmetry-forbidden emission ^5^D_0_ → ^7^F_0_ at 579 nm indicates that the Eu^3+^ ions in **2** occupy sites with low symmetry and have no inversion center.^21^

**Scheme 2:**
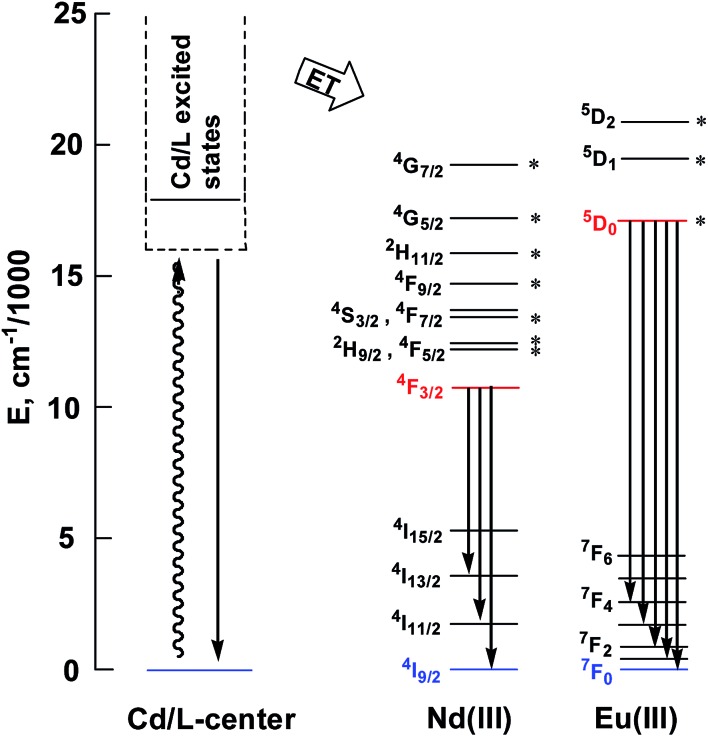
Relevant energy levels in **1–3**. Those marked with * can act as energy acceptors by either Förster or Dexter mechanisms (the former requiring |Δ*J*| = 2, 4, or 6 at the lanthanide, and the latter requiring |Δ*J*| = 0 or 1 with the exception of |Δ*J*| = |Δ*J*′| = 0, which is forbidden).^27^

This is further confirmed by the intensity ratio of 3.9 for *I*(^5^D_0_ → ^7^F_2_)/*I*(^5^D_0_ → ^7^F_1_), which is a good measure of the nature and symmetry of the first coordination sphere of the Eu^3+^ ion.^22^ The overall quantum yield of **2** in CH_3_CN was determined as 0.083 relative to [Ru(bipy)_3_]Cl_2_ in water (bipy = 2,2′-bipyridine; *Φ*_em_ = 0.028)^23^ and corrected for the refractive index of the solvent. **1** and **2** show two excitation bands at approximately 280 nm and 350 nm, while **3** has one at approximately 360 nm, in agreement with their absorption spectra, confirming that the energy transfers from the ligand centers to Ln^3+^ ions occur (Scheme 2). For each Cd–Ln or Ni–Nd nano-cluster the luminescence spectrum in the solid state is similar to that in solution.

For the Nd(iii) nano-clusters **1** and **3**, we were naturally interested in the influence of their structures and d metals on their photophysical properties. The relative emission intensities of **1** and **3** were determined under the same experimental conditions. With the same absorbance value at 355 nm, the relative emission intensity at 1068 nm was estimated to be 5.4 for **1** : **3** in CH_3_CN (Fig. 6), indicating that with the central metal ions encapsulated by chromophoric Cd/L^1^ components (energy transfer donors), **1** has superior luminescence properties compared to **3**. It is also noticeable that, compared to the “enclosed” nano-drum-like structure in **1** the “open” square-like structure of **3** results in metal centers which are less well shielded from outside solvents which may quench lanthanide luminescence.^24^ In addition, the d-block metal ions introduced into the lanthanide-based clusters may play different roles in the luminescence properties of the Ln^3+^ ions. For the Cd^2+^ ion, its saturated d^10^ electronic configuration prevents the quenching of lanthanide luminescence through a d–d transition (f → d energy transfer), while the Ni^2+^ ion may quench the luminescence *via* f → d energy transfer.^25^ The emission lifetimes (*τ*) of **1** and **3** are 1.9 μs and 0.5 μs, respectively. The intrinsic quantum yields (*Φ*_Ln_) of Nd^3+^ emission in **1** and **3** are calculated as 0.76% and 0.20%, respectively, using *Φ*_Ln_ = *τ*/*τ*_0_ (*τ*_0_ = 250 μs, the natural lifetime of Nd^3+^).^26^

**Fig. 7:**
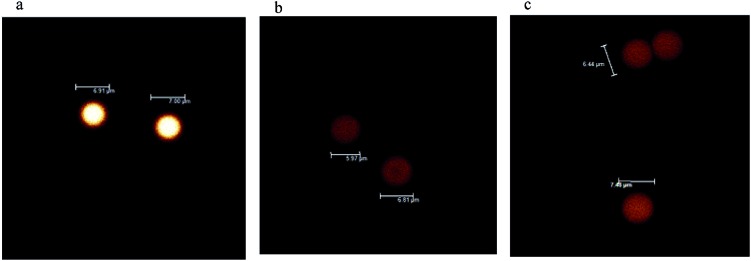
Confocal images of polystyrene beads loaded with Ln–Cd nano-clusters (a) **5**, Nd; (b) **6**, Sm, and (c) **7**, Yb.

To explore the imaging utility of Cd–Ln nano-clusters on an imaging platform used for biological materials, cross-linked polystyrene beads (6–7 μm) containing complexes **5**, **6** and **7** were visualized using confocal microscopy (Fig. 7). Images were collected to show total emission (400–800 nm). All experimental parameters are identical and bead emissions shown are a true representation of brightness on a color scale of black to red to white to blue, where blue indicates saturation. As shown in Fig. 7, beads loaded with **5** are considerably brighter, suggesting that the Nd–Cd nano-cluster may have enhanced emission properties with the H_2_L^2^ Schiff base ligand, compared to the Sm–Cd and Yb–Cd nano-clusters. Further analysis of the relative emission intensities between the bead sets can be undertaken to improve the confidence of this observation.

## Conclusions

Nanostructured materials are increasingly being developed as imaging agents for both fundamental research imaging and diagnostic imaging. Here, we have presented data characterizing a new class of self-assembling lanthanide nano-clusters. These compounds have structures and imaging properties making them suitable candidates for cell and deep tissue imaging agents. Unlike classical nanoparticles, these materials are self-assembling homogeneous particles with asymmetric structures. The Cd–Ln clusters adopt nano-drum-like structures. The Ni–Ln cluster **3** has a square-like architecture. TEM and SAXS data are consistent with these molecular compounds having solution structures similar to those found in the solid state. The different architectures give rise to different imaging properties. With the Ln(iii) centers enclosed within its nano-drum-like structure, Cd–Nd cluster **1** exhibits better NIR luminescence properties than Ni–Nd cluster **3**. The photophysical properties of the nano-drum-like clusters demonstrate their potential as lanthanide emitters, and their utility as bio-imaging agents in confocal microscopy platforms. Future studies will focus on understanding how their molecular structures influence their stability, photophysical properties and ability to be delivered to cells and tissues in nano-formulations.
