# Supplementary material for: Anisotropic lanthanide-based nano-clusters for imaging applications
Source: Faraday Discuss. 2016 Mar 31;191(0):465–79. doi: 10.1039/c6fd00018e (PMC5123638; doi:10.1039/c6fd00018e)
Supplement: Supplementary file 1 [file FD-191-C6FD00018E-s001.pdf]

Electronic Supplementary Material (ESI) for Faraday Discussions.
This journal is © The Royal Society of Chemistry 2016

Supporting Information

Anisotropic Lanthanide-based Nano-clusters for Imaging Applications

Xiaoping Yang, Shiqing Wang, Tyler L. King, Chris Kerr, Clement Blanchet, Dmitri Svergun, Robert Pal, Andrew Beeby, Katherine A. Brown,* Richard A. Jones,* Lijie Zhang and Shaoming Huang*

Contents

1. The ^{1}H NMR spectrum of **1**……...…..……..............………...……….……………………..…..S2

2. Photophysical properties of the free $H_2L^{1,2}$ and clusters.….……………...……….......…..…...S3

3. X-Ray Crystallography…………….………………………….……...…....…....……...…S4

1. **<u>The ^{1}H NMR spectrum of 1</u>**

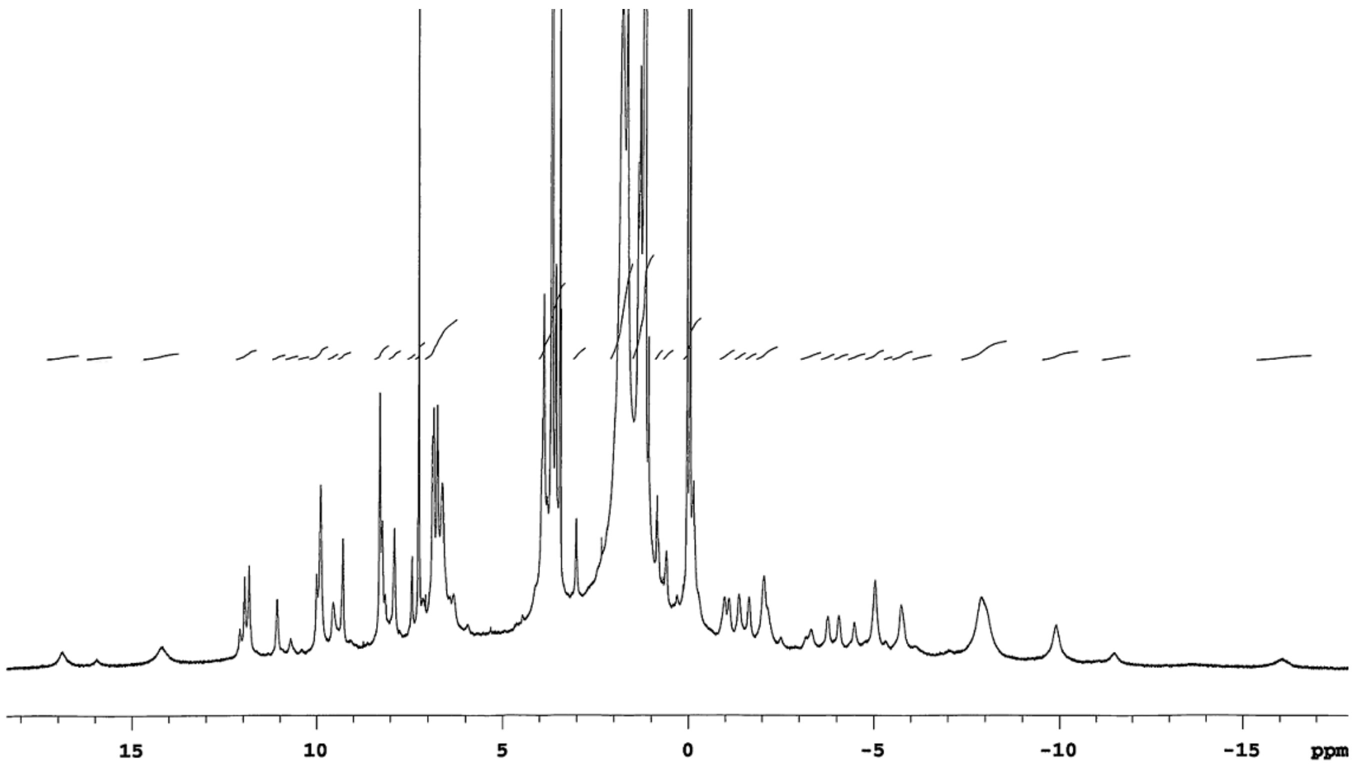

Figure S1. ^{1}H NMR spectrum of **1**.

2. Photophysical properties of the free $H_2L^{1,2}$ and clusters

Figure S2. Excitation and emission spectra of free ligands in CH_3CN.

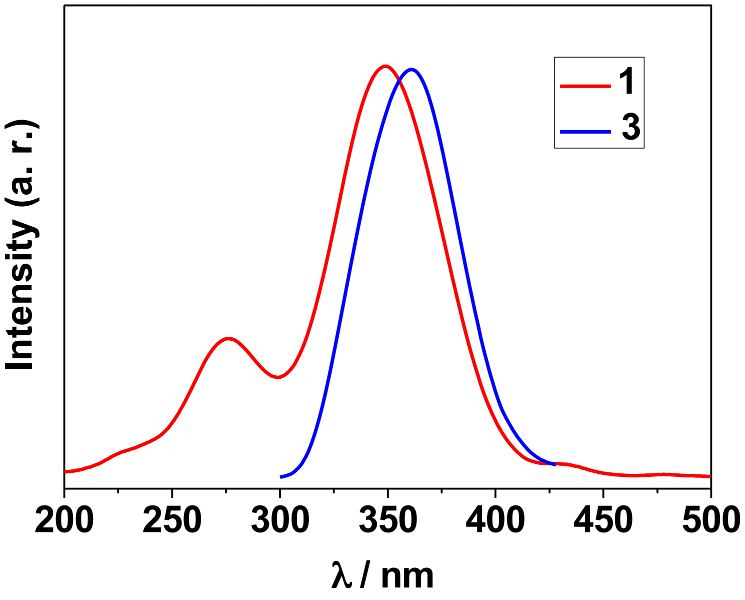

Figure S3. Excitation spectra of **1** and **3** in CH_3CN.

3. X-Ray Crystallography

Table S1. Crystal data and structure refinement for **1-3**.

	**1**	**2**	**3**
Formula	$C_{342}H_{428}Cd_{24}Cl_7Nd_8N_{24}O_{128}$	$C_{342}H_{428}Cd_{24}Cl_7Eu_8N_{24}O_{128}$	$C_{56}H_{50}Br_4Nd_2N_8Ni_4O_{52}$
Fw	11022.75	11084.51	2502.00
Crystal system	Orthorhombic	Orthorhombic	Monoclinic
Space group	Pna2(1)	Pna2(1)	P2(1)/c
a [Å]	54.734(11)	54.106(11)	15.462(3)
b [Å]	27.619(6)	27.127(5)	19.156(4)
c [Å]	35.243(7)	34.876(7)	19.871(4)
α [deg]	90	90	90.00
β [deg]	90	90	107.63(3)
γ [deg]	90	90	90.00
V / [Å^3]	53277(18)	51189(18)	5609.2(19)
d / [g/cm^3]	1.374	1.438	1.481
Z	4	4	2
T [K]	223(1)	223(1)	223(1)
F(000)	21692	21788	2452
μ, mm^{-1}	1.798	2.040	3.073
θ rang, deg	0.94-25.00	2.99-25.00	2.91-25.00
reflns meads	90178	86518	17304
reflns used	90178	86518	9806
params	4762	4762	600
R1^a, wR2^a [$I > 2\sigma(I)$]	0.1018, 0.2608	0.1035, 0.2090	0.0781, 0.2092
R1, wR2 (all data)	0.1213, 0.2844	0.2221, 0.2570	0.0982, 0.2280
Quality of fit	1.038	0.922	1.061

^{a} R1 = $\Sigma|F_o| - |F_c|\Sigma|F_o|$. wR2 = $[\Sigma w[(F_o^2 - F_c^2)^2]/\Sigma|[w(F_o^2)^2]]^{1/2}$. w=1/[$\sigma^2(F_o^2)$+(0.075P)^2], where P = [max(F_o^2,0)+2F_c^2]/3.

Table S2. Selected Bond Lengths (Å) and Angles (°) for **1**.

Nd(1)-O(300)	2.265(14)	Nd(7)-O(319)	2.385(17)
Nd(1)-O(301)	2.321(15)	Nd(7)-O(316)	2.398(15)
Nd(1)-O(46)	2.332(12)	Nd(7)-O(40)	2.610(14)
Nd(1)-O(2)	2.334(16)	Nd(7)-N(18)	2.617(19)
Nd(1)-O(298)	2.348(16)	Nd(8)-O(308)	2.262(15)
Nd(1)-O(265)	2.483(15)	Nd(8)-O(306)	2.345(16)
Nd(1)-O(45)	2.626(12)	Nd(8)-O(309)	2.362(16)
Nd(1)-N(1)	2.65(2)	Nd(8)-O(3)	2.368(18)
Nd(2)-O(10)	2.297(16)	Nd(8)-O(311)	2.371(15)
Nd(2)-O(268)	2.316(14)	Nd(8)-O(47)	2.372(17)
Nd(2)-O(14)	2.336(15)	Nd(8)-O(4)	2.544(19)
Nd(2)-O(270)	2.371(16)	Nd(8)-N(24)	2.59(2)
Nd(2)-O(271)	2.436(18)	Cd(1)-O(302)	2.24(2)
Nd(2)-O(273)	2.464(19)	Cd(1)-O(6)	2.278(17)
Nd(2)-O(9)	2.575(14)	Cd(1)-O(265)	2.285(14)
Nd(2)-N(7)	2.576(17)	Cd(1)-O(2)	2.297(16)
Nd(3)-O(26)	2.327(14)	Cd(1)-N(3)	2.33(3)
Nd(3)-O(280)	2.338(15)	Cd(1)-O(1)	2.575(16)
Nd(3)-O(283)	2.348(14)	Cd(2)-O(6)	2.19(2)
Nd(3)-O(278)	2.353(14)	Cd(2)-O(302)	2.35(2)
Nd(3)-O(281)	2.358(16)	Cd(2)-O(343)	2.359(18)
Nd(3)-O(22)	2.376(12)	Cd(2)-O(5)	2.37(2)
Nd(3)-N(13)	2.527(14)	Cd(2)-Cl(1)	2.385(11)
Nd(3)-O(21)	2.591(15)	Cd(3)-O(269)	2.25(2)
Nd(4)-O(288)	2.277(16)	Cd(3)-N(5)	2.277(16)
Nd(4)-O(34)	2.300(18)	Cd(3)-O(10)	2.315(16)
Nd(4)-O(290)	2.335(18)	Cd(3)-O(267)	2.427(16)
Nd(4)-O(293)	2.398(16)	Cd(3)-O(266)	2.444(13)
Nd(4)-O(291)	2.423(16)	Cd(3)-Cl(1)	2.685(11)
Nd(4)-O(38)	2.428(14)	Cd(4)-O(18)	2.195(16)
Nd(4)-N(19)	2.647(17)	Cd(4)-N(9)	2.238(18)
Nd(4)-O(33)	2.652(17)	Cd(4)-O(272)	2.247(16)
Nd(5)-O(338)	2.268(19)	Cd(4)-O(273)	2.30(2)
Nd(5)-O(15)	2.317(17)	Cd(4)-O(14)	2.349(15)
Nd(5)-O(11)	2.330(18)	Cd(4)-O(13)	2.563(15)
Nd(5)-O(339)	2.371(16)	Cd(5)-O(18)	2.246(14)
Nd(5)-O(336)	2.39(2)	Cd(5)-O(272)	2.326(16)
Nd(5)-O(341)	2.434(16)	Cd(5)-O(275)	2.359(18)
Nd(5)-N(6)	2.57(2)	Cd(5)-O(17)	2.398(15)
Nd(5)-O(16)	2.625(19)	Cd(5)-O(276)	2.437(18)
Nd(6)-O(328)	2.208(14)	Cd(5)-Cl(4)	2.473(6)
Nd(6)-O(23)	2.324(12)	Cd(6)-O(277)	2.244(14)
Nd(6)-O(326)	2.335(15)	Cd(6)-O(279)	2.278(15)
Nd(6)-O(27)	2.338(14)	Cd(6)-N(11)	2.302(17)
Nd(6)-O(331)	2.389(15)	Cd(6)-O(22)	2.345(13)
Nd(6)-O(329)	2.413(15)	Cd(6)-O(276)	2.375(16)
Nd(6)-O(28)	2.592(17)	Cd(6)-O(274)	2.397(13)
Nd(6)-N(12)	2.603(19)	Cd(7)-O(26)	2.245(15)
Nd(7)-O(39)	2.302(14)	Cd(7)-O(30)	2.269(17)
Nd(7)-O(35)	2.362(14)	Cd(7)-N(15)	2.281(16)
Nd(7)-O(318)	2.365(13)	Cd(7)-O(282)	2.302(14)
Nd(7)-O(321)	2.385(16)	Cd(7)-O(283)	2.361(14)

Cd(7)-O(25)	2.511(16)
Cd(8)-O(30)	2.208(15)
Cd(8)-O(286)	2.288(14)
Cd(8)-O(282)	2.315(16)
Cd(8)-O(285)	2.433(19)
Cd(8)-Cl(3)	2.442(12)
Cd(8)-O(29)	2.462(15)
Cd(9)-O(287)	2.230(15)
Cd(9)-O(289)	2.240(17)
Cd(9)-O(34)	2.298(17)
Cd(9)-O(284)	2.323(14)
Cd(9)-N(17)	2.34(2)
Cd(9)-O(285)	2.45(2)
Cd(10)-O(42)	2.233(11)
Cd(10)-O(38)	2.265(15)
Cd(10)-N(21)	2.288(18)
Cd(10)-O(294)	2.289(13)
Cd(10)-O(291)	2.377(13)
Cd(10)-O(37)	2.550(15)
Cd(11)-O(42)	2.268(13)
Cd(11)-O(296)	2.286(15)
Cd(11)-O(294)	2.294(13)
Cd(11)-O(295)	2.404(16)
Cd(11)-Cl(2)	2.446(10)
Cd(11)-O(41)	2.451(17)
Cd(12)-O(297)	2.163(17)
Cd(12)-O(299)	2.249(13)
Cd(12)-N(23)	2.296(17)
Cd(12)-O(46)	2.337(11)
Cd(12)-O(292)	2.398(16)
Cd(12)-O(295)	2.432(15)
Cd(13)-O(335)	2.252(16)
Cd(13)-O(15)	2.278(17)
Cd(13)-O(332)	2.337(16)
Cd(13)-O(333)	2.363(16)
Cd(13)-O(337)	2.39(2)
Cd(13)-N(8)	2.41(2)
Cd(14)-O(19)	2.283(14)
Cd(14)-O(330)	2.330(13)
Cd(14)-O(334)	2.356(19)
Cd(14)-Cl(6)	2.419(7)
Cd(14)-O(20)	2.428(17)
Cd(14)-O(333)	2.430(16)
Cd(15)-O(330)	2.238(13)
Cd(15)-O(19)	2.262(15)
Cd(15)-N(10)	2.300(19)
Cd(15)-O(23)	2.328(13)
Cd(15)-O(331)	2.350(16)
Cd(15)-O(24)	2.518(14)
Cd(16)-O(325)	2.272(15)
Cd(16)-N(14)	2.274(19)
Cd(16)-O(27)	2.307(14)
Cd(16)-O(327)	2.320(11)
Cd(16)-O(322)	2.412(13)
Cd(16)-O(323)	2.413(15)
Cd(17)-O(31)	2.200(14)
Cd(17)-O(324)	2.274(17)
Cd(17)-O(323)	2.348(14)
Cd(17)-O(320)	2.355(15)
Cd(17)-O(32)	2.437(18)
Cd(17)-Cl(5)	2.448(5)
Cd(18)-N(16)	2.22(2)
Cd(18)-O(35)	2.268(15)
Cd(18)-O(31)	2.270(14)
Cd(18)-O(320)	2.295(16)
Cd(18)-O(321)	2.314(12)
Cd(18)-O(36)	2.545(14)
Cd(19)-O(315)	2.250(17)
Cd(19)-O(39)	2.269(14)
Cd(19)-O(317)	2.336(16)
Cd(19)-O(312)	2.349(13)
Cd(19)-N(20)	2.406(19)
Cd(19)-O(313)	2.453(15)
Cd(20)-O(43)	2.313(15)
Cd(20)-O(314)	2.317(18)
Cd(20)-O(310)	2.321(13)
Cd(20)-O(313)	2.335(17)
Cd(20)-Cl(7)	2.466(8)
Cd(20)-O(44)	2.506(16)
Cd(21)-O(43)	2.207(13)
Cd(21)-O(47)	2.211(17)
Cd(21)-O(311)	2.296(15)
Cd(21)-O(310)	2.307(14)
Cd(21)-N(22)	2.315(18)
Cd(21)-O(48)	2.505(19)
Cd(21)-O(312)	2.646(15)
Cd(22)-O(3)	2.264(17)
Cd(22)-N(2)	2.267(15)
Cd(22)-O(305)	2.294(17)
Cd(22)-O(342)	2.329(16)
Cd(22)-O(307)	2.377(18)
Cd(22)-O(304)	2.466(17)
Cd(23)-O(7)	2.246(15)
Cd(23)-O(303)	2.271(16)
Cd(23)-O(340)	2.347(16)
Cd(23)-O(304)	2.396(18)
Cd(23)-O(344)	2.397(17)
Cd(23)-O(8)	2.471(17)
Cd(24)-N(4)	2.21(2)
Cd(24)-O(7)	2.221(18)
Cd(24)-O(340)	2.271(16)
Cd(24)-O(11)	2.272(19)
Cd(24)-O(341)	2.329(15)
Cd(24)-O(12)	2.54(2)
O(300)-Nd(1)-O(301)	142.4(5)
O(300)-Nd(1)-O(46)	83.5(5)
O(301)-Nd(1)-O(46)	110.6(5)
O(300)-Nd(1)-O(2)	121.1(5)

O(301)-Nd(1)-O(2)	78.7(6)
O(46)-Nd(1)-O(2)	127.2(5)
O(300)-Nd(1)-O(298)	75.7(6)
O(301)-Nd(1)-O(298)	73.9(6)
O(46)-Nd(1)-O(298)	77.6(5)
O(2)-Nd(1)-O(298)	148.7(6)
O(300)-Nd(1)-O(265)	80.7(5)
O(301)-Nd(1)-O(265)	77.0(5)
O(46)-Nd(1)-O(265)	161.1(4)
O(2)-Nd(1)-O(265)	70.5(5)
O(298)-Nd(1)-O(265)	88.5(5)
O(300)-Nd(1)-O(45)	142.3(5)
O(301)-Nd(1)-O(45)	70.0(5)
O(46)-Nd(1)-O(45)	62.3(4)
O(2)-Nd(1)-O(45)	74.5(5)
O(298)-Nd(1)-O(45)	109.2(5)
O(265)-Nd(1)-O(45)	135.6(4)
O(300)-Nd(1)-N(1)	77.5(5)
O(301)-Nd(1)-N(1)	139.1(5)
O(46)-Nd(1)-N(1)	73.9(5)
O(2)-Nd(1)-N(1)	68.5(5)
O(298)-Nd(1)-N(1)	142.6(6)
O(265)-Nd(1)-N(1)	112.3(5)
O(45)-Nd(1)-N(1)	78.0(5)
O(10)-Nd(2)-O(268)	80.9(5)
O(10)-Nd(2)-O(14)	131.0(5)
O(268)-Nd(2)-O(14)	117.5(5)
O(10)-Nd(2)-O(270)	80.3(6)
O(268)-Nd(2)-O(270)	75.4(6)
O(14)-Nd(2)-O(270)	145.9(5)
O(10)-Nd(2)-O(271)	111.3(6)
O(268)-Nd(2)-O(271)	146.2(5)
O(14)-Nd(2)-O(271)	79.2(6)
O(270)-Nd(2)-O(271)	75.9(6)
O(10)-Nd(2)-O(273)	157.5(6)
O(268)-Nd(2)-O(273)	81.3(6)
O(14)-Nd(2)-O(273)	70.1(6)
O(270)-Nd(2)-O(273)	82.0(6)
O(271)-Nd(2)-O(273)	77.4(6)
O(10)-Nd(2)-O(9)	66.6(5)
O(268)-Nd(2)-O(9)	141.8(5)
O(14)-Nd(2)-O(9)	74.8(5)
O(270)-Nd(2)-O(9)	115.9(5)
O(271)-Nd(2)-O(9)	68.5(6)
O(273)-Nd(2)-O(9)	134.6(6)
O(10)-Nd(2)-N(7)	71.7(6)
O(268)-Nd(2)-N(7)	74.2(5)
O(14)-Nd(2)-N(7)	71.2(6)
O(270)-Nd(2)-N(7)	141.2(6)
O(271)-Nd(2)-N(7)	139.1(6)
O(273)-Nd(2)-N(7)	116.1(6)
O(9)-Nd(2)-N(7)	76.9(6)
O(26)-Nd(3)-O(280)	118.7(5)
O(26)-Nd(3)-O(283)	69.0(5)
O(280)-Nd(3)-O(283)	79.1(5)
O(26)-Nd(3)-O(278)	146.9(5)
O(280)-Nd(3)-O(278)	75.1(5)
O(283)-Nd(3)-O(278)	86.2(5)
O(26)-Nd(3)-O(281)	78.4(5)
O(280)-Nd(3)-O(281)	142.0(6)
O(283)-Nd(3)-O(281)	76.5(5)
O(278)-Nd(3)-O(281)	74.6(6)
O(26)-Nd(3)-O(22)	128.6(5)
O(280)-Nd(3)-O(22)	86.5(5)
O(283)-Nd(3)-O(22)	161.7(4)
O(278)-Nd(3)-O(22)	79.2(5)
O(281)-Nd(3)-O(22)	109.6(5)
O(26)-Nd(3)-N(13)	73.1(5)
O(280)-Nd(3)-N(13)	76.3(5)
O(283)-Nd(3)-N(13)	115.7(5)
O(278)-Nd(3)-N(13)	139.4(6)
O(281)-Nd(3)-N(13)	141.0(6)
O(22)-Nd(3)-N(13)	70.9(4)
O(26)-Nd(3)-O(21)	74.2(5)
O(280)-Nd(3)-O(21)	144.0(5)
O(283)-Nd(3)-O(21)	134.5(5)
O(278)-Nd(3)-O(21)	113.5(5)
O(281)-Nd(3)-O(21)	70.7(5)
O(22)-Nd(3)-O(21)	62.6(4)
N(13)-Nd(3)-O(21)	76.3(5)
O(288)-Nd(4)-O(34)	77.6(6)
O(288)-Nd(4)-O(290)	76.6(6)
O(34)-Nd(4)-O(290)	86.5(6)
O(288)-Nd(4)-O(293)	76.4(5)
O(34)-Nd(4)-O(293)	110.2(6)
O(290)-Nd(4)-O(293)	144.0(6)
O(288)-Nd(4)-O(291)	87.0(5)
O(34)-Nd(4)-O(291)	160.5(6)
O(290)-Nd(4)-O(291)	78.3(5)
O(293)-Nd(4)-O(291)	77.0(5)
O(288)-Nd(4)-O(38)	150.1(5)
O(34)-Nd(4)-O(38)	127.5(6)
O(290)-Nd(4)-O(38)	116.7(6)
O(293)-Nd(4)-O(38)	79.0(5)
O(291)-Nd(4)-O(38)	71.0(5)
O(288)-Nd(4)-N(19)	141.5(4)
O(34)-Nd(4)-N(19)	72.4(6)
O(290)-Nd(4)-N(19)	78.0(5)
O(293)-Nd(4)-N(19)	136.7(5)
O(291)-Nd(4)-N(19)	115.5(5)
O(38)-Nd(4)-N(19)	68.2(4)
O(288)-Nd(4)-O(33)	107.2(6)
O(34)-Nd(4)-O(33)	60.8(5)
O(290)-Nd(4)-O(33)	144.3(5)
O(293)-Nd(4)-O(33)	67.5(5)
O(291)-Nd(4)-O(33)	136.6(4)
O(38)-Nd(4)-O(33)	78.1(5)
N(19)-Nd(4)-O(33)	78.4(5)

O(338)-Nd(5)-O(15)	84.6(6)
O(338)-Nd(5)-O(11)	116.6(6)
O(15)-Nd(5)-O(11)	130.2(6)
O(338)-Nd(5)-O(339)	145.0(6)
O(15)-Nd(5)-O(339)	107.1(5)
O(11)-Nd(5)-O(339)	81.4(6)
O(338)-Nd(5)-O(336)	74.1(7)
O(15)-Nd(5)-O(336)	75.4(7)
O(11)-Nd(5)-O(336)	151.0(7)
O(339)-Nd(5)-O(336)	77.1(7)
O(338)-Nd(5)-O(341)	81.4(6)
O(15)-Nd(5)-O(341)	159.4(6)
O(11)-Nd(5)-O(341)	70.0(6)
O(339)-Nd(5)-O(341)	77.2(6)
O(336)-Nd(5)-O(341)	86.2(6)
O(338)-Nd(5)-N(6)	74.2(7)
O(15)-Nd(5)-N(6)	73.1(6)
O(11)-Nd(5)-N(6)	71.1(7)
O(339)-Nd(5)-N(6)	140.5(7)
O(336)-Nd(5)-N(6)	136.8(7)
O(341)-Nd(5)-N(6)	116.8(5)
O(338)-Nd(5)-O(16)	142.8(6)
O(15)-Nd(5)-O(16)	63.1(6)
O(11)-Nd(5)-O(16)	76.9(6)
O(339)-Nd(5)-O(16)	67.4(6)
O(336)-Nd(5)-O(16)	111.6(6)
O(341)-Nd(5)-O(16)	134.5(5)
N(6)-Nd(5)-O(16)	78.9(6)
O(328)-Nd(6)-O(23)	117.3(5)
O(328)-Nd(6)-O(326)	72.5(5)
O(23)-Nd(6)-O(326)	151.5(5)
O(328)-Nd(6)-O(27)	86.3(5)
O(23)-Nd(6)-O(27)	129.0(5)
O(326)-Nd(6)-O(27)	76.1(5)
O(328)-Nd(6)-O(331)	78.2(5)
O(23)-Nd(6)-O(331)	71.8(5)
O(326)-Nd(6)-O(331)	85.3(5)
O(27)-Nd(6)-O(331)	158.7(5)
O(328)-Nd(6)-O(329)	139.6(5)
O(23)-Nd(6)-O(329)	82.6(5)
O(326)-Nd(6)-O(329)	75.2(5)
O(27)-Nd(6)-O(329)	108.8(5)
O(331)-Nd(6)-O(329)	75.7(5)
O(328)-Nd(6)-O(28)	144.9(6)
O(23)-Nd(6)-O(28)	75.7(5)
O(326)-Nd(6)-O(28)	112.5(5)
O(27)-Nd(6)-O(28)	63.1(5)
O(331)-Nd(6)-O(28)	135.7(6)
O(329)-Nd(6)-O(28)	70.9(6)
O(328)-Nd(6)-N(12)	77.0(6)
O(23)-Nd(6)-N(12)	70.3(5)
O(326)-Nd(6)-N(12)	137.3(5)
O(27)-Nd(6)-N(12)	72.7(5)
O(331)-Nd(6)-N(12)	116.9(5)

O(329)-Nd(6)-N(12)	142.8(5)
O(28)-Nd(6)-N(12)	77.8(6)
O(39)-Nd(7)-O(35)	128.8(5)
O(39)-Nd(7)-O(318)	84.1(5)
O(35)-Nd(7)-O(318)	122.6(5)
O(39)-Nd(7)-O(321)	161.5(5)
O(35)-Nd(7)-O(321)	69.6(5)
O(318)-Nd(7)-O(321)	83.5(5)
O(39)-Nd(7)-O(319)	107.6(5)
O(35)-Nd(7)-O(319)	78.4(5)
O(318)-Nd(7)-O(319)	142.3(5)
O(321)-Nd(7)-O(319)	75.0(5)
O(39)-Nd(7)-O(316)	78.7(6)
O(35)-Nd(7)-O(316)	145.6(5)
O(318)-Nd(7)-O(316)	74.7(5)
O(321)-Nd(7)-O(316)	84.9(5)
O(319)-Nd(7)-O(316)	72.9(5)
O(39)-Nd(7)-O(40)	62.5(5)
O(35)-Nd(7)-O(40)	73.2(5)
O(318)-Nd(7)-O(40)	141.9(5)
O(321)-Nd(7)-O(40)	133.1(5)
O(319)-Nd(7)-O(40)	70.3(5)
O(316)-Nd(7)-O(40)	112.9(6)
O(39)-Nd(7)-N(18)	74.2(6)
O(35)-Nd(7)-N(18)	72.8(6)
O(318)-Nd(7)-N(18)	75.1(5)
O(321)-Nd(7)-N(18)	115.4(6)
O(319)-Nd(7)-N(18)	142.2(5)
O(316)-Nd(7)-N(18)	141.0(6)
O(40)-Nd(7)-N(18)	78.3(5)
O(308)-Nd(8)-O(306)	76.0(5)
O(308)-Nd(8)-O(309)	146.5(6)
O(306)-Nd(8)-O(309)	73.0(6)
O(308)-Nd(8)-O(3)	83.5(6)
O(306)-Nd(8)-O(3)	78.9(6)
O(309)-Nd(8)-O(3)	102.4(6)
O(308)-Nd(8)-O(311)	81.7(5)
O(306)-Nd(8)-O(311)	80.6(5)
O(309)-Nd(8)-O(311)	81.1(6)
O(3)-Nd(8)-O(311)	157.0(6)
O(308)-Nd(8)-O(47)	116.4(6)
O(306)-Nd(8)-O(47)	142.3(5)
O(309)-Nd(8)-O(47)	82.5(6)
O(3)-Nd(8)-O(47)	135.4(6)
O(311)-Nd(8)-O(47)	67.4(6)
O(308)-Nd(8)-O(4)	139.9(5)
O(306)-Nd(8)-O(4)	116.2(6)
O(309)-Nd(8)-O(4)	67.7(6)
O(3)-Nd(8)-O(4)	63.7(5)
O(311)-Nd(8)-O(4)	136.2(5)
O(47)-Nd(8)-O(4)	78.4(6)
O(308)-Nd(8)-N(24)	75.3(7)
O(306)-Nd(8)-N(24)	142.2(6)
O(309)-Nd(8)-N(24)	138.2(7)

O(3)-Nd(8)-N(24)	74.0(7)
O(311)-Nd(8)-N(24)	118.5(7)
O(47)-Nd(8)-N(24)	73.7(6)
O(4)-Nd(8)-N(24)	74.0(7)
O(302)-Cd(1)-O(6)	78.6(7)
O(302)-Cd(1)-O(265)	90.9(6)
O(6)-Cd(1)-O(265)	137.5(6)
O(302)-Cd(1)-O(2)	98.0(7)
O(6)-Cd(1)-O(2)	147.0(5)
O(265)-Cd(1)-O(2)	74.8(5)
O(302)-Cd(1)-N(3)	158.3(8)
O(6)-Cd(1)-N(3)	81.2(9)
O(265)-Cd(1)-N(3)	109.5(7)
O(2)-Cd(1)-N(3)	94.6(9)
O(302)-Cd(1)-O(1)	83.3(7)
O(6)-Cd(1)-O(1)	79.4(6)
O(265)-Cd(1)-O(1)	140.7(6)
O(2)-Cd(1)-O(1)	67.7(6)
N(3)-Cd(1)-O(1)	85.2(8)
O(6)-Cd(2)-O(302)	78.0(7)
O(6)-Cd(2)-O(343)	133.1(7)
O(302)-Cd(2)-O(343)	104.3(7)
O(6)-Cd(2)-O(5)	69.7(7)
O(302)-Cd(2)-O(5)	146.8(8)
O(343)-Cd(2)-O(5)	92.2(7)
O(6)-Cd(2)-Cl(1)	100.3(5)
O(302)-Cd(2)-Cl(1)	95.6(6)
O(343)-Cd(2)-Cl(1)	125.5(7)
O(5)-Cd(2)-Cl(1)	97.9(6)
O(269)-Cd(3)-N(5)	163.3(6)
O(269)-Cd(3)-O(10)	114.5(7)
N(5)-Cd(3)-O(10)	78.4(6)
O(269)-Cd(3)-O(267)	93.2(7)
N(5)-Cd(3)-O(267)	97.0(6)
O(10)-Cd(3)-O(267)	91.9(6)
O(269)-Cd(3)-O(266)	80.2(6)
N(5)-Cd(3)-O(266)	84.5(5)
O(10)-Cd(3)-O(266)	157.7(5)
O(267)-Cd(3)-O(266)	104.4(5)
O(269)-Cd(3)-Cl(1)	86.0(6)
N(5)-Cd(3)-Cl(1)	86.2(5)
O(10)-Cd(3)-Cl(1)	79.1(5)
O(267)-Cd(3)-Cl(1)	169.6(5)
O(266)-Cd(3)-Cl(1)	85.7(4)
O(18)-Cd(4)-N(9)	81.8(6)
O(18)-Cd(4)-O(272)	76.2(5)
N(9)-Cd(4)-O(272)	156.3(7)
O(18)-Cd(4)-O(273)	135.8(6)
N(9)-Cd(4)-O(273)	111.3(7)
O(272)-Cd(4)-O(273)	90.8(6)
O(18)-Cd(4)-O(14)	150.3(5)
N(9)-Cd(4)-O(14)	96.1(6)
O(272)-Cd(4)-O(14)	98.7(5)
O(273)-Cd(4)-O(14)	72.7(6)
O(18)-Cd(4)-O(13)	84.7(5)
N(9)-Cd(4)-O(13)	81.3(6)
O(272)-Cd(4)-O(13)	88.0(5)
O(273)-Cd(4)-O(13)	137.7(6)
O(14)-Cd(4)-O(13)	65.7(5)
O(18)-Cd(5)-O(272)	73.6(6)
O(18)-Cd(5)-O(275)	149.7(5)
O(272)-Cd(5)-O(275)	114.5(6)
O(18)-Cd(5)-O(17)	69.6(6)
O(272)-Cd(5)-O(17)	143.2(6)
O(275)-Cd(5)-O(17)	97.0(6)
O(18)-Cd(5)-O(276)	95.2(5)
O(272)-Cd(5)-O(276)	92.2(6)
O(275)-Cd(5)-O(276)	56.6(6)
O(17)-Cd(5)-O(276)	89.8(5)
O(18)-Cd(5)-Cl(4)	108.2(4)
O(272)-Cd(5)-Cl(4)	101.1(4)
O(275)-Cd(5)-Cl(4)	98.9(4)
O(17)-Cd(5)-Cl(4)	91.7(4)
O(276)-Cd(5)-Cl(4)	155.4(4)
O(277)-Cd(6)-O(279)	90.3(6)
O(277)-Cd(6)-N(11)	172.3(6)
O(279)-Cd(6)-N(11)	93.5(6)
O(277)-Cd(6)-O(22)	110.0(5)
O(279)-Cd(6)-O(22)	95.5(5)
N(11)-Cd(6)-O(22)	76.3(5)
O(277)-Cd(6)-O(276)	86.7(6)
O(279)-Cd(6)-O(276)	176.8(6)
N(11)-Cd(6)-O(276)	89.6(6)
O(22)-Cd(6)-O(276)	84.5(5)
O(277)-Cd(6)-O(274)	87.5(5)
O(279)-Cd(6)-O(274)	97.3(5)
N(11)-Cd(6)-O(274)	85.5(5)
O(22)-Cd(6)-O(274)	158.3(4)
O(276)-Cd(6)-O(274)	83.6(6)
O(26)-Cd(7)-O(30)	146.4(6)
O(26)-Cd(7)-N(15)	97.0(6)
O(30)-Cd(7)-N(15)	85.8(6)
O(26)-Cd(7)-O(282)	95.2(5)
O(30)-Cd(7)-O(282)	76.0(5)
N(15)-Cd(7)-O(282)	161.0(6)
O(26)-Cd(7)-O(283)	70.1(5)
O(30)-Cd(7)-O(283)	141.3(5)
N(15)-Cd(7)-O(283)	106.1(6)
O(282)-Cd(7)-O(283)	91.8(5)
O(26)-Cd(7)-O(25)	67.1(5)
O(30)-Cd(7)-O(25)	79.8(6)
N(15)-Cd(7)-O(25)	85.1(6)
O(282)-Cd(7)-O(25)	86.3(5)
O(283)-Cd(7)-O(25)	136.8(5)
O(30)-Cd(8)-O(286)	147.0(7)
O(30)-Cd(8)-O(282)	76.9(6)
O(286)-Cd(8)-O(282)	106.5(5)
O(30)-Cd(8)-O(285)	94.0(6)

O(286)-Cd(8)-O(285)	54.8(6)
O(282)-Cd(8)-O(285)	83.8(6)
O(30)-Cd(8)-Cl(3)	111.7(5)
O(286)-Cd(8)-Cl(3)	99.6(5)
O(282)-Cd(8)-Cl(3)	103.5(5)
O(285)-Cd(8)-Cl(3)	154.2(5)
O(30)-Cd(8)-O(29)	67.6(5)
O(286)-Cd(8)-O(29)	103.7(5)
O(282)-Cd(8)-O(29)	144.5(5)
O(285)-Cd(8)-O(29)	98.9(6)
Cl(3)-Cd(8)-O(29)	89.3(5)
O(287)-Cd(9)-O(289)	94.6(6)
O(287)-Cd(9)-O(34)	104.4(6)
O(289)-Cd(9)-O(34)	97.1(6)
O(287)-Cd(9)-O(284)	89.0(5)
O(289)-Cd(9)-O(284)	94.6(6)
O(34)-Cd(9)-O(284)	161.4(6)
O(287)-Cd(9)-N(17)	168.9(8)
O(289)-Cd(9)-N(17)	93.9(7)
O(34)-Cd(9)-N(17)	81.6(6)
O(284)-Cd(9)-N(17)	83.1(6)
O(287)-Cd(9)-O(285)	81.0(6)
O(289)-Cd(9)-O(285)	175.7(6)
O(34)-Cd(9)-O(285)	84.2(6)
O(284)-Cd(9)-O(285)	85.3(6)
N(17)-Cd(9)-O(285)	90.4(7)
O(42)-Cd(10)-O(38)	149.0(5)
O(42)-Cd(10)-N(21)	83.4(6)
O(38)-Cd(10)-N(21)	93.5(6)
O(42)-Cd(10)-O(294)	75.7(5)
O(38)-Cd(10)-O(294)	101.8(5)
N(21)-Cd(10)-O(294)	158.6(5)
O(42)-Cd(10)-O(291)	135.9(5)
O(38)-Cd(10)-O(291)	74.7(5)
N(21)-Cd(10)-O(291)	105.1(6)
O(294)-Cd(10)-O(291)	93.5(5)
O(42)-Cd(10)-O(37)	83.8(4)
O(38)-Cd(10)-O(37)	65.3(5)
N(21)-Cd(10)-O(37)	88.0(6)
O(294)-Cd(10)-O(37)	84.9(4)
O(291)-Cd(10)-O(37)	138.6(5)
O(42)-Cd(11)-O(296)	149.5(6)
O(42)-Cd(11)-O(294)	74.9(5)
O(296)-Cd(11)-O(294)	117.9(6)
O(42)-Cd(11)-O(295)	102.4(5)
O(296)-Cd(11)-O(295)	55.2(5)
O(294)-Cd(11)-O(295)	84.6(5)
O(42)-Cd(11)-Cl(2)	112.6(4)
O(296)-Cd(11)-Cl(2)	93.3(5)
O(294)-Cd(11)-Cl(2)	99.4(4)
O(295)-Cd(11)-Cl(2)	144.6(4)
O(42)-Cd(11)-O(41)	68.4(4)
O(296)-Cd(11)-O(41)	95.5(6)
O(294)-Cd(11)-O(41)	143.3(5)

O(295)-Cd(11)-O(41)	104.8(6)
Cl(2)-Cd(11)-O(41)	92.8(5)
O(297)-Cd(12)-O(299)	91.8(6)
O(297)-Cd(12)-N(23)	171.0(6)
O(299)-Cd(12)-N(23)	96.0(5)
O(297)-Cd(12)-O(46)	105.3(5)
O(299)-Cd(12)-O(46)	95.6(4)
N(23)-Cd(12)-O(46)	78.5(5)
O(297)-Cd(12)-O(292)	92.7(6)
O(299)-Cd(12)-O(292)	96.1(5)
N(23)-Cd(12)-O(292)	82.0(6)
O(46)-Cd(12)-O(292)	158.2(5)
O(297)-Cd(12)-O(295)	86.2(6)
O(299)-Cd(12)-O(295)	177.9(6)
N(23)-Cd(12)-O(295)	86.0(5)
O(46)-Cd(12)-O(295)	85.5(4)
O(292)-Cd(12)-O(295)	83.4(5)
O(335)-Cd(13)-O(15)	107.3(6)
O(335)-Cd(13)-O(332)	87.3(5)
O(15)-Cd(13)-O(332)	160.7(6)
O(335)-Cd(13)-O(333)	82.9(6)
O(15)-Cd(13)-O(333)	84.9(5)
O(332)-Cd(13)-O(333)	84.4(5)
O(335)-Cd(13)-O(337)	93.0(6)
O(15)-Cd(13)-O(337)	98.1(6)
O(332)-Cd(13)-O(337)	93.6(6)
O(333)-Cd(13)-O(337)	175.5(6)
O(335)-Cd(13)-N(8)	168.6(7)
O(15)-Cd(13)-N(8)	77.5(5)
O(332)-Cd(13)-N(8)	85.9(5)
O(333)-Cd(13)-N(8)	87.4(6)
O(337)-Cd(13)-N(8)	96.5(6)
O(19)-Cd(14)-O(330)	75.4(5)
O(19)-Cd(14)-O(334)	145.0(5)
O(330)-Cd(14)-O(334)	116.9(6)
O(19)-Cd(14)-Cl(6)	115.1(4)
O(330)-Cd(14)-Cl(6)	99.9(4)
O(334)-Cd(14)-Cl(6)	95.8(5)
O(19)-Cd(14)-O(20)	67.7(6)
O(330)-Cd(14)-O(20)	142.8(5)
O(334)-Cd(14)-O(20)	97.0(6)
Cl(6)-Cd(14)-O(20)	91.1(4)
O(19)-Cd(14)-O(333)	94.7(5)
O(330)-Cd(14)-O(333)	86.6(5)
O(334)-Cd(14)-O(333)	56.1(6)
Cl(6)-Cd(14)-O(333)	150.2(4)
O(20)-Cd(14)-O(333)	101.2(5)
O(330)-Cd(15)-O(19)	77.7(5)
O(330)-Cd(15)-N(10)	161.3(6)
O(19)-Cd(15)-N(10)	83.8(6)
O(330)-Cd(15)-O(23)	99.9(5)
O(19)-Cd(15)-O(23)	149.0(5)
N(10)-Cd(15)-O(23)	94.6(6)
O(330)-Cd(15)-O(331)	92.6(5)

O(19)-Cd(15)-O(331)	138.2(5)
N(10)-Cd(15)-O(331)	103.0(6)
O(23)-Cd(15)-O(331)	72.4(5)
O(330)-Cd(15)-O(24)	86.4(5)
O(19)-Cd(15)-O(24)	82.4(5)
N(10)-Cd(15)-O(24)	88.6(6)
O(23)-Cd(15)-O(24)	66.5(4)
O(331)-Cd(15)-O(24)	138.1(5)
O(325)-Cd(16)-N(14)	163.9(6)
O(325)-Cd(16)-O(27)	110.4(5)
N(14)-Cd(16)-O(27)	79.0(6)
O(325)-Cd(16)-O(327)	91.8(5)
N(14)-Cd(16)-O(327)	100.1(6)
O(27)-Cd(16)-O(327)	97.0(5)
O(325)-Cd(16)-O(322)	86.4(5)
N(14)-Cd(16)-O(322)	81.5(6)
O(27)-Cd(16)-O(322)	157.6(5)
O(327)-Cd(16)-O(322)	97.0(4)
O(325)-Cd(16)-O(323)	82.6(5)
N(14)-Cd(16)-O(323)	85.5(6)
O(27)-Cd(16)-O(323)	84.2(5)
O(327)-Cd(16)-O(323)	174.3(5)
O(322)-Cd(16)-O(323)	83.6(5)
O(31)-Cd(17)-O(324)	149.9(5)
O(31)-Cd(17)-O(323)	95.8(5)
O(324)-Cd(17)-O(323)	56.1(5)
O(31)-Cd(17)-O(320)	76.1(5)
O(324)-Cd(17)-O(320)	110.7(6)
O(323)-Cd(17)-O(320)	89.6(5)
O(31)-Cd(17)-O(32)	69.0(6)
O(324)-Cd(17)-O(32)	101.8(6)
O(323)-Cd(17)-O(32)	97.8(6)
O(320)-Cd(17)-O(32)	144.9(6)
O(31)-Cd(17)-Cl(5)	113.4(3)
O(324)-Cd(17)-Cl(5)	94.1(4)
O(323)-Cd(17)-Cl(5)	150.2(4)
O(320)-Cd(17)-Cl(5)	102.6(4)
O(32)-Cd(17)-Cl(5)	87.8(4)
N(16)-Cd(18)-O(35)	97.4(7)
N(16)-Cd(18)-O(31)	82.9(7)
O(35)-Cd(18)-O(31)	151.3(4)
N(16)-Cd(18)-O(320)	157.1(6)
O(35)-Cd(18)-O(320)	97.1(6)
O(31)-Cd(18)-O(320)	75.9(5)
N(16)-Cd(18)-O(321)	107.5(6)
O(35)-Cd(18)-O(321)	72.5(5)
O(31)-Cd(18)-O(321)	135.0(5)
O(320)-Cd(18)-O(321)	93.7(5)
N(16)-Cd(18)-O(36)	83.5(6)
O(35)-Cd(18)-O(36)	65.2(5)
O(31)-Cd(18)-O(36)	86.4(5)
O(320)-Cd(18)-O(36)	86.6(5)
O(321)-Cd(18)-O(36)	137.3(5)
O(315)-Cd(19)-O(39)	108.5(5)
O(315)-Cd(19)-O(317)	88.2(6)
O(39)-Cd(19)-O(317)	92.6(5)
O(315)-Cd(19)-O(312)	87.7(5)
O(39)-Cd(19)-O(312)	158.8(5)
O(317)-Cd(19)-O(312)	101.8(5)
O(315)-Cd(19)-N(20)	171.1(6)
O(39)-Cd(19)-N(20)	80.3(6)
O(317)-Cd(19)-N(20)	92.2(6)
O(312)-Cd(19)-N(20)	83.5(5)
O(315)-Cd(19)-O(313)	84.6(6)
O(39)-Cd(19)-O(313)	81.8(5)
O(317)-Cd(19)-O(313)	168.9(5)
O(312)-Cd(19)-O(313)	86.3(5)
N(20)-Cd(19)-O(313)	96.2(6)
O(43)-Cd(20)-O(314)	141.2(7)
O(43)-Cd(20)-O(310)	76.2(5)
O(314)-Cd(20)-O(310)	119.0(6)
O(43)-Cd(20)-O(313)	92.7(5)
O(314)-Cd(20)-O(313)	55.5(6)
O(310)-Cd(20)-O(313)	88.2(5)
O(43)-Cd(20)-Cl(7)	123.1(5)
O(314)-Cd(20)-Cl(7)	93.8(5)
O(310)-Cd(20)-Cl(7)	90.1(4)
O(313)-Cd(20)-Cl(7)	142.6(4)
O(43)-Cd(20)-O(44)	68.0(4)
O(314)-Cd(20)-O(44)	95.6(6)
O(310)-Cd(20)-O(44)	142.9(5)
O(313)-Cd(20)-O(44)	102.5(5)
Cl(7)-Cd(20)-O(44)	101.2(4)
O(43)-Cd(21)-O(47)	147.8(6)
O(43)-Cd(21)-O(311)	139.7(6)
O(47)-Cd(21)-O(311)	71.4(5)
O(43)-Cd(21)-O(310)	78.6(5)
O(47)-Cd(21)-O(310)	100.2(5)
O(311)-Cd(21)-O(310)	86.1(5)
O(43)-Cd(21)-N(22)	83.4(6)
O(47)-Cd(21)-N(22)	93.1(6)
O(311)-Cd(21)-N(22)	110.3(5)
O(310)-Cd(21)-N(22)	161.7(5)
O(43)-Cd(21)-O(48)	81.6(6)
O(47)-Cd(21)-O(48)	66.2(6)
O(311)-Cd(21)-O(48)	135.3(6)
O(310)-Cd(21)-O(48)	88.1(5)
N(22)-Cd(21)-O(48)	85.8(6)
O(43)-Cd(21)-O(312)	88.0(5)
O(47)-Cd(21)-O(312)	123.9(5)
O(311)-Cd(21)-O(312)	52.6(5)
O(310)-Cd(21)-O(312)	81.1(5)
N(22)-Cd(21)-O(312)	101.9(5)
O(48)-Cd(21)-O(312)	166.3(4)
O(3)-Cd(22)-N(2)	78.1(6)
O(3)-Cd(22)-O(305)	111.1(6)
N(2)-Cd(22)-O(305)	167.4(7)
O(3)-Cd(22)-O(342)	157.7(6)

N(2)-Cd(22)-O(342)	84.0(6)	O(7)-Cd(23)-O(8)	67.7(6)
O(305)-Cd(22)-O(342)	84.9(6)	O(303)-Cd(23)-O(8)	97.6(6)
O(3)-Cd(22)-O(307)	98.1(6)	O(340)-Cd(23)-O(8)	142.8(6)
N(2)-Cd(22)-O(307)	96.0(6)	O(304)-Cd(23)-O(8)	94.6(6)
O(305)-Cd(22)-O(307)	91.4(7)	O(344)-Cd(23)-O(8)	94.2(6)
O(342)-Cd(22)-O(307)	97.0(6)	N(4)-Cd(24)-O(7)	83.0(8)
O(3)-Cd(22)-O(304)	82.6(6)	N(4)-Cd(24)-O(340)	159.7(8)
N(2)-Cd(22)-O(304)	88.0(6)	O(7)-Cd(24)-O(340)	77.1(5)
O(305)-Cd(22)-O(304)	84.7(7)	N(4)-Cd(24)-O(11)	96.5(8)
O(342)-Cd(22)-O(304)	83.5(6)	O(7)-Cd(24)-O(11)	149.8(7)
O(307)-Cd(22)-O(304)	176.1(6)	O(340)-Cd(24)-O(11)	98.8(6)
O(7)-Cd(23)-O(303)	143.9(7)	N(4)-Cd(24)-O(341)	111.1(8)
O(7)-Cd(23)-O(340)	75.1(6)	O(7)-Cd(24)-O(341)	135.5(6)
O(303)-Cd(23)-O(340)	113.8(6)	O(340)-Cd(24)-O(341)	86.2(5)
O(7)-Cd(23)-O(304)	91.9(6)	O(11)-Cd(24)-O(341)	72.9(6)
O(303)-Cd(23)-O(304)	55.3(6)	N(4)-Cd(24)-O(12)	84.5(8)
O(340)-Cd(23)-O(304)	88.0(6)	O(7)-Cd(24)-O(12)	83.2(6)
O(7)-Cd(23)-O(344)	116.4(6)	O(340)-Cd(24)-O(12)	89.5(6)
O(303)-Cd(23)-O(344)	96.7(6)	O(11)-Cd(24)-O(12)	66.7(7)
O(340)-Cd(23)-O(344)	101.0(6)	O(341)-Cd(24)-O(12)	138.2(6)
O(304)-Cd(23)-O(344)	151.6(5)		

Table S3. Selected Bond Lengths (Å) and Angles (°) for **2**.

Eu(1)-O(300)	2.260(17)	Eu(7)-O(321)	2.366(18)
Eu(1)-O(46)	2.283(17)	Eu(7)-O(319)	2.418(19)
Eu(1)-O(298)	2.339(18)	Eu(7)-N(18)	2.53(2)
Eu(1)-O(265)	2.375(18)	Eu(7)-O(40)	2.642(18)
Eu(1)-O(301)	2.387(18)	Eu(8)-O(308)	2.228(19)
Eu(1)-O(2)	2.43(2)	Eu(8)-O(309)	2.267(19)
Eu(1)-O(45)	2.629(18)	Eu(8)-O(311)	2.272(18)
Eu(1)-N(1)	2.66(2)	Eu(8)-O(47)	2.312(18)
Eu(2)-O(268)	2.257(19)	Eu(8)-O(306)	2.38(2)
Eu(2)-O(271)	2.304(18)	Eu(8)-O(3)	2.39(2)
Eu(2)-O(10)	2.317(19)	Eu(8)-N(24)	2.56(3)
Eu(2)-O(14)	2.358(19)	Eu(8)-O(4)	2.59(2)
Eu(2)-O(270)	2.369(18)	Cd(1)-O(302)	2.16(2)
Eu(2)-O(273)	2.480(19)	Cd(1)-N(3)	2.16(3)
Eu(2)-N(7)	2.53(2)	Cd(1)-O(6)	2.19(2)
Eu(2)-O(9)	2.58(2)	Cd(1)-O(2)	2.25(2)
Eu(3)-O(26)	2.271(18)	Cd(1)-O(265)	2.272(18)
Eu(3)-O(280)	2.274(18)	Cd(1)-O(1)	2.63(2)
Eu(3)-O(278)	2.313(17)	Cd(2)-O(6)	2.12(2)
Eu(3)-O(281)	2.321(18)	Cd(2)-O(5)	2.38(2)
Eu(3)-O(22)	2.353(17)	Cd(2)-O(302)	2.38(2)
Eu(3)-O(283)	2.46(2)	Cd(2)-O(343)	2.39(2)
Eu(3)-O(21)	2.52(2)	Cd(2)-Cl(1)	2.476(14)
Eu(3)-N(13)	2.54(2)	Cd(3)-O(269)	2.23(2)
Eu(4)-O(290)	2.26(2)	Cd(3)-N(5)	2.29(3)
Eu(4)-O(34)	2.266(19)	Cd(3)-O(267)	2.31(2)
Eu(4)-O(288)	2.28(2)	Cd(3)-O(10)	2.344(19)
Eu(4)-O(38)	2.296(18)	Cd(3)-O(266)	2.413(16)
Eu(4)-O(291)	2.398(19)	Cd(3)-Cl(1)	2.692(14)
Eu(4)-O(293)	2.414(19)	Cd(4)-O(18)	2.163(17)
Eu(4)-N(19)	2.64(2)	Cd(4)-N(9)	2.23(2)
Eu(4)-O(33)	2.67(2)	Cd(4)-O(272)	2.235(18)
Eu(5)-O(338)	2.14(2)	Cd(4)-O(273)	2.251(19)
Eu(5)-O(11)	2.276(18)	Cd(4)-O(14)	2.365(19)
Eu(5)-O(339)	2.29(2)	Cd(4)-O(13)	2.523(19)
Eu(5)-O(15)	2.35(2)	Cd(5)-O(18)	2.200(17)
Eu(5)-O(341)	2.35(2)	Cd(5)-O(272)	2.206(19)
Eu(5)-O(336)	2.41(2)	Cd(5)-O(275)	2.25(2)
Eu(5)-N(6)	2.55(3)	Cd(5)-O(17)	2.368(19)
Eu(5)-O(16)	2.55(2)	Cd(5)-O(276)	2.438(18)
Eu(6)-O(328)	2.247(16)	Cd(5)-Cl(4)	2.462(8)
Eu(6)-O(27)	2.329(17)	Cd(6)-O(277)	2.21(2)
Eu(6)-O(326)	2.340(17)	Cd(6)-O(22)	2.264(17)
Eu(6)-O(329)	2.350(17)	Cd(6)-N(11)	2.29(2)
Eu(6)-O(23)	2.384(16)	Cd(6)-O(279)	2.293(18)
Eu(6)-O(331)	2.479(18)	Cd(6)-O(274)	2.324(18)
Eu(6)-O(28)	2.586(18)	Cd(6)-O(276)	2.352(18)
Eu(6)-N(12)	2.65(2)	Cd(7)-N(15)	2.20(2)
Eu(7)-O(316)	2.273(18)	Cd(7)-O(30)	2.202(19)
Eu(7)-O(39)	2.293(19)	Cd(7)-O(283)	2.269(19)
Eu(7)-O(318)	2.294(16)	Cd(7)-O(26)	2.272(19)
Eu(7)-O(35)	2.329(17)	Cd(7)-O(282)	2.295(18)

Cd(7)-O(25)	2.45(2)
Cd(7)-O(284)	2.613(18)
Cd(8)-O(30)	2.158(18)
Cd(8)-O(286)	2.25(2)
Cd(8)-O(282)	2.350(19)
Cd(8)-O(285)	2.405(19)
Cd(8)-O(29)	2.421(19)
Cd(8)-Cl(3)	2.438(11)
Cd(9)-O(289)	2.245(17)
Cd(9)-O(34)	2.25(2)
Cd(9)-O(287)	2.254(18)
Cd(9)-N(17)	2.27(2)
Cd(9)-O(284)	2.333(18)
Cd(9)-O(285)	2.399(19)
Cd(10)-N(21)	2.20(2)
Cd(10)-O(294)	2.232(17)
Cd(10)-O(42)	2.251(15)
Cd(10)-O(38)	2.267(17)
Cd(10)-O(291)	2.311(19)
Cd(10)-O(37)	2.544(19)
Cd(11)-O(42)	2.221(15)
Cd(11)-O(296)	2.30(2)
Cd(11)-O(294)	2.338(16)
Cd(11)-O(295)	2.377(18)
Cd(11)-O(41)	2.444(19)
Cd(11)-Cl(2)	2.460(10)
Cd(12)-O(297)	2.241(18)
Cd(12)-N(23)	2.27(2)
Cd(12)-O(292)	2.295(18)
Cd(12)-O(46)	2.324(17)
Cd(12)-O(299)	2.355(19)
Cd(12)-O(295)	2.441(17)
Cd(13)-O(335)	2.17(2)
Cd(13)-O(15)	2.239(19)
Cd(13)-O(337)	2.26(2)
Cd(13)-O(332)	2.311(18)
Cd(13)-N(8)	2.39(3)
Cd(13)-O(333)	2.41(2)
Cd(14)-O(19)	2.284(19)
Cd(14)-O(330)	2.292(19)
Cd(14)-O(334)	2.30(3)
Cd(14)-O(20)	2.377(19)
Cd(14)-O(333)	2.40(2)
Cd(14)-Cl(6)	2.408(9)
Cd(14)-C(334)	2.76(4)
Cd(15)-O(330)	2.244(18)
Cd(15)-N(10)	2.25(2)
Cd(15)-O(19)	2.266(19)
Cd(15)-O(331)	2.291(18)
Cd(15)-O(23)	2.298(15)
Cd(15)-O(24)	2.581(18)
Cd(16)-N(14)	2.23(2)
Cd(16)-O(327)	2.277(18)
Cd(16)-O(325)	2.286(17)
Cd(16)-O(27)	2.317(18)
Cd(16)-O(322)	2.360(18)
Cd(16)-O(323)	2.416(18)
Cd(17)-O(31)	2.154(19)
Cd(17)-O(324)	2.238(19)
Cd(17)-O(320)	2.322(19)
Cd(17)-O(323)	2.351(18)
Cd(17)-Cl(5)	2.469(8)
Cd(17)-O(32)	2.52(2)
Cd(18)-N(16)	2.20(2)
Cd(18)-O(320)	2.210(19)
Cd(18)-O(31)	2.270(19)
Cd(18)-O(35)	2.301(19)
Cd(18)-O(321)	2.331(17)
Cd(18)-O(36)	2.541(18)
Cd(19)-O(315)	2.211(16)
Cd(19)-O(317)	2.309(17)
Cd(19)-N(20)	2.33(2)
Cd(19)-O(39)	2.346(19)
Cd(19)-O(312)	2.385(19)
Cd(19)-O(313)	2.51(2)
Cd(20)-O(313)	2.28(2)
Cd(20)-O(310)	2.286(19)
Cd(20)-O(43)	2.29(2)
Cd(20)-O(314)	2.295(19)
Cd(20)-O(44)	2.394(19)
Cd(20)-Cl(7)	2.411(11)
Cd(21)-O(43)	2.214(19)
Cd(21)-O(47)	2.238(18)
Cd(21)-N(22)	2.24(2)
Cd(21)-O(310)	2.272(19)
Cd(21)-O(311)	2.380(18)
Cd(21)-O(48)	2.49(2)
Cd(21)-O(312)	2.60(2)
Cd(22)-O(305)	2.25(2)
Cd(22)-N(2)	2.26(2)
Cd(22)-O(3)	2.27(2)
Cd(22)-O(307)	2.27(2)
Cd(22)-O(342)	2.305(19)
Cd(22)-O(304)	2.44(2)
Cd(23)-O(7)	2.261(19)
Cd(23)-O(303)	2.29(2)
Cd(23)-O(340)	2.30(2)
Cd(23)-O(344)	2.416(19)
Cd(23)-O(8)	2.44(2)
Cd(23)-O(304)	2.46(2)
Cd(24)-O(7)	2.10(2)
Cd(24)-N(4)	2.28(3)
Cd(24)-O(11)	2.28(2)
Cd(24)-O(341)	2.30(2)
Cd(24)-O(340)	2.33(2)
Cd(24)-O(12)	2.53(2)
Cd(24)-O(342)	2.648(19)
O(300)-Eu(1)-O(46)	85.0(6)

O(300)-Eu(1)-O(298)	76.5(6)
O(46)-Eu(1)-O(298)	78.8(6)
O(300)-Eu(1)-O(265)	79.8(6)
O(46)-Eu(1)-O(265)	161.6(6)
O(298)-Eu(1)-O(265)	87.6(6)
O(300)-Eu(1)-O(301)	141.3(6)
O(46)-Eu(1)-O(301)	111.0(6)
O(298)-Eu(1)-O(301)	72.8(6)
O(265)-Eu(1)-O(301)	75.9(6)
O(300)-Eu(1)-O(2)	118.6(7)
O(46)-Eu(1)-O(2)	127.8(7)
O(298)-Eu(1)-O(2)	148.0(7)
O(265)-Eu(1)-O(2)	69.4(7)
O(301)-Eu(1)-O(2)	80.0(7)
O(300)-Eu(1)-O(45)	144.7(6)
O(46)-Eu(1)-O(45)	63.2(6)
O(298)-Eu(1)-O(45)	109.6(6)
O(265)-Eu(1)-O(45)	134.0(6)
O(301)-Eu(1)-O(45)	69.7(6)
O(2)-Eu(1)-O(45)	75.2(7)
O(300)-Eu(1)-N(1)	76.5(7)
O(46)-Eu(1)-N(1)	72.3(7)
O(298)-Eu(1)-N(1)	141.8(7)
O(265)-Eu(1)-N(1)	113.6(7)
O(301)-Eu(1)-N(1)	141.2(7)
O(2)-Eu(1)-N(1)	69.9(8)
O(45)-Eu(1)-N(1)	79.2(6)
O(268)-Eu(2)-O(271)	147.4(7)
O(268)-Eu(2)-O(10)	84.2(7)
O(271)-Eu(2)-O(10)	108.2(7)
O(268)-Eu(2)-O(14)	115.6(7)
O(271)-Eu(2)-O(14)	80.4(7)
O(10)-Eu(2)-O(14)	129.3(7)
O(268)-Eu(2)-O(270)	76.7(7)
O(271)-Eu(2)-O(270)	75.8(6)
O(10)-Eu(2)-O(270)	80.6(6)
O(14)-Eu(2)-O(270)	146.9(6)
O(268)-Eu(2)-O(273)	82.1(7)
O(271)-Eu(2)-O(273)	77.3(6)
O(10)-Eu(2)-O(273)	160.5(7)
O(14)-Eu(2)-O(273)	69.6(6)
O(270)-Eu(2)-O(273)	82.7(6)
O(268)-Eu(2)-N(7)	75.5(7)
O(271)-Eu(2)-N(7)	136.9(7)
O(10)-Eu(2)-N(7)	70.3(6)
O(14)-Eu(2)-N(7)	70.8(6)
O(270)-Eu(2)-N(7)	141.3(6)
O(273)-Eu(2)-N(7)	119.1(7)
O(268)-Eu(2)-O(9)	138.4(7)
O(271)-Eu(2)-O(9)	70.6(7)
O(10)-Eu(2)-O(9)	60.7(7)
O(14)-Eu(2)-O(9)	77.3(7)
O(270)-Eu(2)-O(9)	115.0(7)
O(273)-Eu(2)-O(9)	137.0(7)
N(7)-Eu(2)-O(9)	72.2(7)
O(26)-Eu(3)-O(280)	118.0(6)
O(26)-Eu(3)-O(278)	147.8(7)
O(280)-Eu(3)-O(278)	75.0(6)
O(26)-Eu(3)-O(281)	78.5(6)
O(280)-Eu(3)-O(281)	142.4(7)
O(278)-Eu(3)-O(281)	75.5(6)
O(26)-Eu(3)-O(22)	130.0(6)
O(280)-Eu(3)-O(22)	84.7(6)
O(278)-Eu(3)-O(22)	77.9(6)
O(281)-Eu(3)-O(22)	111.2(6)
O(26)-Eu(3)-O(283)	68.7(6)
O(280)-Eu(3)-O(283)	80.4(6)
O(278)-Eu(3)-O(283)	86.3(6)
O(281)-Eu(3)-O(283)	74.9(6)
O(22)-Eu(3)-O(283)	160.6(6)
O(26)-Eu(3)-O(21)	73.7(6)
O(280)-Eu(3)-O(21)	146.1(7)
O(278)-Eu(3)-O(21)	112.9(6)
O(281)-Eu(3)-O(21)	68.4(7)
O(22)-Eu(3)-O(21)	66.2(6)
O(283)-Eu(3)-O(21)	131.5(6)
O(26)-Eu(3)-N(13)	71.6(7)
O(280)-Eu(3)-N(13)	75.6(6)
O(278)-Eu(3)-N(13)	139.7(7)
O(281)-Eu(3)-N(13)	140.9(7)
O(22)-Eu(3)-N(13)	72.5(6)
O(283)-Eu(3)-N(13)	115.0(6)
O(21)-Eu(3)-N(13)	79.2(7)
O(290)-Eu(4)-O(34)	84.8(7)
O(290)-Eu(4)-O(288)	73.8(7)
O(34)-Eu(4)-O(288)	77.9(7)
O(290)-Eu(4)-O(38)	119.9(7)
O(34)-Eu(4)-O(38)	129.5(7)
O(288)-Eu(4)-O(38)	147.7(6)
O(290)-Eu(4)-O(291)	79.6(6)
O(34)-Eu(4)-O(291)	159.7(7)
O(288)-Eu(4)-O(291)	85.3(7)
O(38)-Eu(4)-O(291)	70.2(6)
O(290)-Eu(4)-O(293)	141.3(7)
O(34)-Eu(4)-O(293)	110.0(7)
O(288)-Eu(4)-O(293)	74.8(7)
O(38)-Eu(4)-O(293)	78.8(6)
O(291)-Eu(4)-O(293)	75.8(6)
O(290)-Eu(4)-N(19)	79.7(7)
O(34)-Eu(4)-N(19)	73.8(7)
O(288)-Eu(4)-N(19)	142.6(7)
O(38)-Eu(4)-N(19)	69.2(6)
O(291)-Eu(4)-N(19)	115.5(7)
O(293)-Eu(4)-N(19)	138.2(7)
O(290)-Eu(4)-O(33)	143.5(6)
O(34)-Eu(4)-O(33)	61.0(6)
O(288)-Eu(4)-O(33)	108.2(7)
O(38)-Eu(4)-O(33)	78.7(6)

O(291)-Eu(4)-O(33)	136.5(6)
O(293)-Eu(4)-O(33)	68.9(6)
N(19)-Eu(4)-O(33)	78.7(7)
O(338)-Eu(5)-O(11)	117.4(7)
O(338)-Eu(5)-O(339)	142.6(8)
O(11)-Eu(5)-O(339)	81.5(7)
O(338)-Eu(5)-O(15)	85.3(7)
O(11)-Eu(5)-O(15)	130.3(7)
O(339)-Eu(5)-O(15)	107.3(7)
O(338)-Eu(5)-O(341)	80.2(7)
O(11)-Eu(5)-O(341)	70.2(7)
O(339)-Eu(5)-O(341)	76.6(7)
O(15)-Eu(5)-O(341)	159.1(7)
O(338)-Eu(5)-O(336)	74.0(7)
O(11)-Eu(5)-O(336)	152.3(7)
O(339)-Eu(5)-O(336)	76.4(7)
O(15)-Eu(5)-O(336)	73.0(7)
O(341)-Eu(5)-O(336)	88.5(7)
O(338)-Eu(5)-N(6)	74.8(8)
O(11)-Eu(5)-N(6)	71.8(8)
O(339)-Eu(5)-N(6)	142.2(8)
O(15)-Eu(5)-N(6)	73.2(8)
O(341)-Eu(5)-N(6)	116.7(8)
O(336)-Eu(5)-N(6)	135.3(8)
O(338)-Eu(5)-O(16)	146.1(7)
O(11)-Eu(5)-O(16)	74.5(7)
O(339)-Eu(5)-O(16)	67.0(7)
O(15)-Eu(5)-O(16)	65.6(7)
O(341)-Eu(5)-O(16)	132.2(7)
O(336)-Eu(5)-O(16)	110.9(7)
N(6)-Eu(5)-O(16)	80.1(8)
O(328)-Eu(6)-O(27)	87.4(6)
O(328)-Eu(6)-O(326)	75.7(6)
O(27)-Eu(6)-O(326)	79.3(6)
O(328)-Eu(6)-O(329)	139.8(6)
O(27)-Eu(6)-O(329)	110.9(6)
O(326)-Eu(6)-O(329)	73.3(6)
O(328)-Eu(6)-O(23)	115.3(6)
O(27)-Eu(6)-O(23)	127.3(6)
O(326)-Eu(6)-O(23)	149.7(5)
O(329)-Eu(6)-O(23)	82.4(6)
O(328)-Eu(6)-O(331)	77.9(6)
O(27)-Eu(6)-O(331)	160.6(6)
O(326)-Eu(6)-O(331)	84.9(6)
O(329)-Eu(6)-O(331)	74.5(6)
O(23)-Eu(6)-O(331)	71.1(6)
O(328)-Eu(6)-O(28)	146.6(6)
O(27)-Eu(6)-O(28)	63.5(6)
O(326)-Eu(6)-O(28)	111.9(5)
O(329)-Eu(6)-O(28)	70.5(5)
O(23)-Eu(6)-O(28)	75.3(5)
O(331)-Eu(6)-O(28)	133.9(6)
O(328)-Eu(6)-N(12)	75.9(6)
O(27)-Eu(6)-N(12)	70.7(6)
O(326)-Eu(6)-N(12)	139.1(6)
O(329)-Eu(6)-N(12)	143.3(6)
O(23)-Eu(6)-N(12)	70.4(6)
O(331)-Eu(6)-N(12)	116.8(6)
O(28)-Eu(6)-N(12)	78.9(6)
O(316)-Eu(7)-O(39)	82.6(7)
O(316)-Eu(7)-O(318)	75.5(6)
O(39)-Eu(7)-O(318)	85.1(6)
O(316)-Eu(7)-O(35)	145.1(7)
O(39)-Eu(7)-O(35)	126.0(7)
O(318)-Eu(7)-O(35)	121.6(6)
O(316)-Eu(7)-O(321)	83.4(6)
O(39)-Eu(7)-O(321)	162.9(6)
O(318)-Eu(7)-O(321)	82.0(6)
O(35)-Eu(7)-O(321)	70.8(6)
O(316)-Eu(7)-O(319)	72.8(6)
O(39)-Eu(7)-O(319)	110.3(6)
O(318)-Eu(7)-O(319)	142.3(6)
O(35)-Eu(7)-O(319)	77.8(6)
O(321)-Eu(7)-O(319)	74.5(6)
O(316)-Eu(7)-N(18)	143.4(7)
O(39)-Eu(7)-N(18)	69.9(7)
O(318)-Eu(7)-N(18)	78.4(6)
O(35)-Eu(7)-N(18)	71.2(7)
O(321)-Eu(7)-N(18)	117.9(6)
O(319)-Eu(7)-N(18)	138.8(6)
O(316)-Eu(7)-O(40)	112.7(6)
O(39)-Eu(7)-O(40)	60.2(6)
O(318)-Eu(7)-O(40)	141.4(6)
O(35)-Eu(7)-O(40)	74.1(6)
O(321)-Eu(7)-O(40)	135.1(6)
O(319)-Eu(7)-O(40)	71.5(6)
N(18)-Eu(7)-O(40)	74.4(6)
O(308)-Eu(8)-O(309)	146.1(7)
O(308)-Eu(8)-O(311)	79.0(6)
O(309)-Eu(8)-O(311)	81.5(6)
O(308)-Eu(8)-O(47)	115.3(7)
O(309)-Eu(8)-O(47)	82.9(7)
O(311)-Eu(8)-O(47)	69.9(6)
O(308)-Eu(8)-O(306)	77.1(7)
O(309)-Eu(8)-O(306)	72.3(7)
O(311)-Eu(8)-O(306)	79.4(7)
O(47)-Eu(8)-O(306)	143.0(7)
O(308)-Eu(8)-O(3)	84.6(7)
O(309)-Eu(8)-O(3)	103.4(6)
O(311)-Eu(8)-O(3)	155.8(7)
O(47)-Eu(8)-O(3)	133.9(7)
O(306)-Eu(8)-O(3)	79.6(7)
O(308)-Eu(8)-N(24)	76.1(8)
O(309)-Eu(8)-N(24)	137.7(8)
O(311)-Eu(8)-N(24)	118.4(7)
O(47)-Eu(8)-N(24)	71.8(7)
O(306)-Eu(8)-N(24)	143.8(7)
O(3)-Eu(8)-N(24)	74.0(7)

O(308)-Eu(8)-O(4)	140.2(7)
O(309)-Eu(8)-O(4)	68.7(7)
O(311)-Eu(8)-O(4)	138.6(7)
O(47)-Eu(8)-O(4)	78.3(7)
O(306)-Eu(8)-O(4)	115.6(7)
O(3)-Eu(8)-O(4)	62.9(7)
N(24)-Eu(8)-O(4)	73.3(7)
O(302)-Cd(1)-N(3)	160.8(9)
O(302)-Cd(1)-O(6)	79.5(8)
N(3)-Cd(1)-O(6)	82.8(10)
O(302)-Cd(1)-O(2)	97.6(8)
N(3)-Cd(1)-O(2)	92.1(9)
O(6)-Cd(1)-O(2)	143.4(8)
O(302)-Cd(1)-O(265)	90.4(7)
N(3)-Cd(1)-O(265)	108.2(9)
O(6)-Cd(1)-O(265)	141.4(7)
O(2)-Cd(1)-O(265)	74.4(7)
O(302)-Cd(1)-O(1)	82.8(7)
N(3)-Cd(1)-O(1)	86.8(8)
O(6)-Cd(1)-O(1)	80.5(8)
O(2)-Cd(1)-O(1)	63.1(8)
O(265)-Cd(1)-O(1)	135.5(7)
O(6)-Cd(2)-O(5)	71.9(9)
O(6)-Cd(2)-O(302)	76.2(8)
O(5)-Cd(2)-O(302)	147.9(8)
O(6)-Cd(2)-O(343)	130.7(8)
O(5)-Cd(2)-O(343)	92.8(8)
O(302)-Cd(2)-O(343)	105.1(8)
O(6)-Cd(2)-Cl(1)	97.1(7)
O(5)-Cd(2)-Cl(1)	93.4(6)
O(302)-Cd(2)-Cl(1)	94.3(6)
O(343)-Cd(2)-Cl(1)	131.1(7)
O(269)-Cd(3)-N(5)	166.1(9)
O(269)-Cd(3)-O(267)	89.3(7)
N(5)-Cd(3)-O(267)	100.0(9)
O(269)-Cd(3)-O(10)	112.7(7)
N(5)-Cd(3)-O(10)	77.3(9)
O(267)-Cd(3)-O(10)	93.0(7)
O(269)-Cd(3)-O(266)	81.5(6)
N(5)-Cd(3)-O(266)	86.2(9)
O(267)-Cd(3)-O(266)	104.5(6)
O(10)-Cd(3)-O(266)	157.8(6)
O(269)-Cd(3)-Cl(1)	87.4(6)
N(5)-Cd(3)-Cl(1)	85.7(8)
O(267)-Cd(3)-Cl(1)	167.0(6)
O(10)-Cd(3)-Cl(1)	76.7(6)
O(266)-Cd(3)-Cl(1)	87.5(5)
O(18)-Cd(4)-N(9)	81.5(7)
O(18)-Cd(4)-O(272)	72.3(7)
N(9)-Cd(4)-O(272)	152.8(8)
O(18)-Cd(4)-O(273)	134.4(7)
N(9)-Cd(4)-O(273)	111.6(8)
O(272)-Cd(4)-O(273)	92.2(7)
O(18)-Cd(4)-O(14)	150.5(6)

N(9)-Cd(4)-O(14)	97.7(7)
O(272)-Cd(4)-O(14)	101.7(7)
O(273)-Cd(4)-O(14)	73.5(7)
O(18)-Cd(4)-O(13)	86.0(6)
N(9)-Cd(4)-O(13)	83.5(7)
O(272)-Cd(4)-O(13)	87.7(6)
O(273)-Cd(4)-O(13)	137.2(7)
O(14)-Cd(4)-O(13)	64.7(7)
O(18)-Cd(5)-O(272)	72.1(7)
O(18)-Cd(5)-O(275)	148.2(7)
O(272)-Cd(5)-O(275)	115.8(8)
O(18)-Cd(5)-O(17)	70.2(6)
O(272)-Cd(5)-O(17)	142.3(7)
O(275)-Cd(5)-O(17)	96.1(7)
O(18)-Cd(5)-O(276)	95.1(6)
O(272)-Cd(5)-O(276)	91.4(6)
O(275)-Cd(5)-O(276)	55.5(7)
O(17)-Cd(5)-O(276)	90.7(6)
O(18)-Cd(5)-Cl(4)	107.1(5)
O(272)-Cd(5)-Cl(4)	101.8(5)
O(275)-Cd(5)-Cl(4)	101.3(6)
O(17)-Cd(5)-Cl(4)	90.4(5)
O(276)-Cd(5)-Cl(4)	156.7(5)
O(277)-Cd(6)-O(22)	108.4(7)
O(277)-Cd(6)-N(11)	172.4(7)
O(22)-Cd(6)-N(11)	78.5(7)
O(277)-Cd(6)-O(279)	89.8(7)
O(22)-Cd(6)-O(279)	94.6(6)
N(11)-Cd(6)-O(279)	92.9(7)
O(277)-Cd(6)-O(274)	88.3(7)
O(22)-Cd(6)-O(274)	159.1(6)
N(11)-Cd(6)-O(274)	84.3(7)
O(279)-Cd(6)-O(274)	98.0(6)
O(277)-Cd(6)-O(276)	87.0(7)
O(22)-Cd(6)-O(276)	85.7(6)
N(11)-Cd(6)-O(276)	90.5(7)
O(279)-Cd(6)-O(276)	176.6(6)
O(274)-Cd(6)-O(276)	82.6(6)
N(15)-Cd(7)-O(30)	86.2(8)
N(15)-Cd(7)-O(283)	104.8(8)
O(30)-Cd(7)-O(283)	135.9(7)
N(15)-Cd(7)-O(26)	98.6(8)
O(30)-Cd(7)-O(26)	149.4(7)
O(283)-Cd(7)-O(26)	72.2(7)
N(15)-Cd(7)-O(282)	161.3(8)
O(30)-Cd(7)-O(282)	75.5(7)
O(283)-Cd(7)-O(282)	91.4(6)
O(26)-Cd(7)-O(282)	95.2(7)
N(15)-Cd(7)-O(25)	87.3(8)
O(30)-Cd(7)-O(25)	81.1(7)
O(283)-Cd(7)-O(25)	140.7(7)
O(26)-Cd(7)-O(25)	69.1(7)
O(282)-Cd(7)-O(25)	85.9(7)
N(15)-Cd(7)-O(284)	100.2(7)

O(30)-Cd(7)-O(284)	86.5(6)
O(283)-Cd(7)-O(284)	49.9(6)
O(26)-Cd(7)-O(284)	121.8(6)
O(282)-Cd(7)-O(284)	83.0(6)
O(25)-Cd(7)-O(284)	165.1(6)
O(30)-Cd(8)-O(286)	142.9(7)
O(30)-Cd(8)-O(282)	75.2(7)
O(286)-Cd(8)-O(282)	104.7(7)
O(30)-Cd(8)-O(285)	88.5(7)
O(286)-Cd(8)-O(285)	55.0(7)
O(282)-Cd(8)-O(285)	84.7(6)
O(30)-Cd(8)-O(29)	69.7(7)
O(286)-Cd(8)-O(29)	105.9(7)
O(282)-Cd(8)-O(29)	144.6(6)
O(285)-Cd(8)-O(29)	98.5(7)
O(30)-Cd(8)-Cl(3)	117.3(6)
O(286)-Cd(8)-Cl(3)	99.2(6)
O(282)-Cd(8)-Cl(3)	102.8(5)
O(285)-Cd(8)-Cl(3)	154.2(5)
O(29)-Cd(8)-Cl(3)	89.6(5)
O(289)-Cd(9)-O(34)	96.9(7)
O(289)-Cd(9)-O(287)	91.9(6)
O(34)-Cd(9)-O(287)	105.8(7)
O(289)-Cd(9)-N(17)	93.6(7)
O(34)-Cd(9)-N(17)	81.7(8)
O(287)-Cd(9)-N(17)	170.1(8)
O(289)-Cd(9)-O(284)	97.3(6)
O(34)-Cd(9)-O(284)	161.0(7)
O(287)-Cd(9)-O(284)	86.4(6)
N(17)-Cd(9)-O(284)	84.8(7)
O(289)-Cd(9)-O(285)	177.5(6)
O(34)-Cd(9)-O(285)	82.5(7)
O(287)-Cd(9)-O(285)	86.0(7)
N(17)-Cd(9)-O(285)	88.7(7)
O(284)-Cd(9)-O(285)	83.9(6)
N(21)-Cd(10)-O(294)	161.4(7)
N(21)-Cd(10)-O(42)	85.5(7)
O(294)-Cd(10)-O(42)	76.5(6)
N(21)-Cd(10)-O(38)	93.7(7)
O(294)-Cd(10)-O(38)	99.8(6)
O(42)-Cd(10)-O(38)	151.0(6)
N(21)-Cd(10)-O(291)	102.3(7)
O(294)-Cd(10)-O(291)	94.0(6)
O(42)-Cd(10)-O(291)	136.3(6)
O(38)-Cd(10)-O(291)	72.3(7)
N(21)-Cd(10)-O(37)	89.5(7)
O(294)-Cd(10)-O(37)	84.0(6)
O(42)-Cd(10)-O(37)	83.3(6)
O(38)-Cd(10)-O(37)	67.7(6)
O(291)-Cd(10)-O(37)	138.9(6)
O(42)-Cd(11)-O(296)	149.8(7)
O(42)-Cd(11)-O(294)	75.0(6)
O(296)-Cd(11)-O(294)	113.5(7)
O(42)-Cd(11)-O(295)	99.3(6)
O(296)-Cd(11)-O(295)	55.7(6)
O(294)-Cd(11)-O(295)	82.6(6)
O(42)-Cd(11)-O(41)	66.5(6)
O(296)-Cd(11)-O(41)	102.9(7)
O(294)-Cd(11)-O(41)	141.1(6)
O(295)-Cd(11)-O(41)	108.4(6)
O(42)-Cd(11)-Cl(2)	115.5(5)
O(296)-Cd(11)-Cl(2)	92.2(6)
O(294)-Cd(11)-Cl(2)	100.1(5)
O(295)-Cd(11)-Cl(2)	144.7(5)
O(41)-Cd(11)-Cl(2)	91.6(5)
O(297)-Cd(12)-N(23)	166.2(7)
O(297)-Cd(12)-O(292)	88.6(6)
N(23)-Cd(12)-O(292)	80.4(7)
O(297)-Cd(12)-O(46)	107.4(6)
N(23)-Cd(12)-O(46)	81.5(7)
O(292)-Cd(12)-O(46)	158.6(6)
O(297)-Cd(12)-O(299)	93.1(7)
N(23)-Cd(12)-O(299)	96.6(7)
O(292)-Cd(12)-O(299)	97.4(6)
O(46)-Cd(12)-O(299)	95.8(6)
O(297)-Cd(12)-O(295)	85.4(6)
N(23)-Cd(12)-O(295)	84.8(7)
O(292)-Cd(12)-O(295)	81.7(6)
O(46)-Cd(12)-O(295)	85.5(5)
O(299)-Cd(12)-O(295)	178.2(6)
O(335)-Cd(13)-O(15)	108.4(7)
O(335)-Cd(13)-O(337)	91.6(8)
O(15)-Cd(13)-O(337)	100.3(7)
O(335)-Cd(13)-O(332)	86.1(7)
O(15)-Cd(13)-O(332)	158.9(7)
O(337)-Cd(13)-O(332)	94.4(7)
O(335)-Cd(13)-N(8)	168.3(8)
O(15)-Cd(13)-N(8)	76.9(8)
O(337)-Cd(13)-N(8)	97.7(8)
O(332)-Cd(13)-N(8)	86.2(8)
O(335)-Cd(13)-O(333)	83.1(7)
O(15)-Cd(13)-O(333)	82.5(7)
O(337)-Cd(13)-O(333)	174.6(8)
O(332)-Cd(13)-O(333)	84.1(7)
N(8)-Cd(13)-O(333)	87.4(8)
O(19)-Cd(14)-O(330)	76.3(7)
O(19)-Cd(14)-O(334)	146.6(8)
O(330)-Cd(14)-O(334)	115.2(8)
O(19)-Cd(14)-O(20)	66.4(7)
O(330)-Cd(14)-O(20)	142.1(7)
O(334)-Cd(14)-O(20)	99.9(8)
O(19)-Cd(14)-O(333)	97.5(7)
O(330)-Cd(14)-O(333)	87.1(7)
O(334)-Cd(14)-O(333)	54.5(8)
O(20)-Cd(14)-O(333)	103.4(7)
O(19)-Cd(14)-Cl(6)	113.5(5)
O(330)-Cd(14)-Cl(6)	99.1(5)
O(334)-Cd(14)-Cl(6)	96.2(7)

O(20)-Cd(14)-Cl(6)	90.3(5)
O(333)-Cd(14)-Cl(6)	149.0(6)
O(330)-Cd(15)-N(10)	161.6(7)
O(330)-Cd(15)-O(19)	77.6(7)
N(10)-Cd(15)-O(19)	84.0(7)
O(330)-Cd(15)-O(331)	94.2(7)
N(10)-Cd(15)-O(331)	100.3(7)
O(19)-Cd(15)-O(331)	137.4(7)
O(330)-Cd(15)-O(23)	101.1(6)
N(10)-Cd(15)-O(23)	93.5(7)
O(19)-Cd(15)-O(23)	146.3(6)
O(331)-Cd(15)-O(23)	76.1(6)
O(330)-Cd(15)-O(24)	85.4(6)
N(10)-Cd(15)-O(24)	90.8(7)
O(19)-Cd(15)-O(24)	82.1(6)
O(331)-Cd(15)-O(24)	139.5(6)
O(23)-Cd(15)-O(24)	64.3(6)
N(14)-Cd(16)-O(327)	98.1(7)
N(14)-Cd(16)-O(325)	165.5(7)
O(327)-Cd(16)-O(325)	92.1(6)
N(14)-Cd(16)-O(27)	77.2(7)
O(327)-Cd(16)-O(27)	95.8(6)
O(325)-Cd(16)-O(27)	112.2(6)
N(14)-Cd(16)-O(322)	83.1(7)
O(327)-Cd(16)-O(322)	95.9(7)
O(325)-Cd(16)-O(322)	85.5(6)
O(27)-Cd(16)-O(322)	158.3(6)
N(14)-Cd(16)-O(323)	86.9(7)
O(327)-Cd(16)-O(323)	174.9(6)
O(325)-Cd(16)-O(323)	83.1(6)
O(27)-Cd(16)-O(323)	84.4(6)
O(322)-Cd(16)-O(323)	85.5(6)
O(31)-Cd(17)-O(324)	153.1(7)
O(31)-Cd(17)-O(320)	73.8(7)
O(324)-Cd(17)-O(320)	112.3(7)
O(31)-Cd(17)-O(323)	99.0(6)
O(324)-Cd(17)-O(323)	55.7(6)
O(320)-Cd(17)-O(323)	91.7(6)
O(31)-Cd(17)-Cl(5)	112.7(5)
O(324)-Cd(17)-Cl(5)	91.5(5)
O(320)-Cd(17)-Cl(5)	105.1(5)
O(323)-Cd(17)-Cl(5)	147.1(5)
O(31)-Cd(17)-O(32)	67.1(7)
O(324)-Cd(17)-O(32)	104.6(7)
O(320)-Cd(17)-O(32)	140.6(6)
O(323)-Cd(17)-O(32)	98.1(7)
Cl(5)-Cd(17)-O(32)	86.8(5)
N(16)-Cd(18)-O(320)	158.3(8)
N(16)-Cd(18)-O(31)	86.7(8)
O(320)-Cd(18)-O(31)	73.8(7)
N(16)-Cd(18)-O(35)	94.3(8)
O(320)-Cd(18)-O(35)	97.9(7)
O(31)-Cd(18)-O(35)	150.1(6)
N(16)-Cd(18)-O(321)	106.7(7)
O(320)-Cd(18)-O(321)	94.1(6)
O(31)-Cd(18)-O(321)	136.3(6)
O(35)-Cd(18)-O(321)	71.9(6)
N(16)-Cd(18)-O(36)	82.9(7)
O(320)-Cd(18)-O(36)	85.7(6)
O(31)-Cd(18)-O(36)	84.6(6)
O(35)-Cd(18)-O(36)	66.0(6)
O(321)-Cd(18)-O(36)	137.4(6)
O(315)-Cd(19)-O(317)	89.8(6)
O(315)-Cd(19)-N(20)	170.2(7)
O(317)-Cd(19)-N(20)	90.2(7)
O(315)-Cd(19)-O(39)	111.9(6)
O(317)-Cd(19)-O(39)	94.7(6)
N(20)-Cd(19)-O(39)	77.9(7)
O(315)-Cd(19)-O(312)	85.7(7)
O(317)-Cd(19)-O(312)	98.3(6)
N(20)-Cd(19)-O(312)	84.7(7)
O(39)-Cd(19)-O(312)	158.2(7)
O(315)-Cd(19)-O(313)	83.7(6)
O(317)-Cd(19)-O(313)	170.0(6)
N(20)-Cd(19)-O(313)	97.6(7)
O(39)-Cd(19)-O(313)	80.8(6)
O(312)-Cd(19)-O(313)	88.8(6)
O(313)-Cd(20)-O(310)	90.8(7)
O(313)-Cd(20)-O(43)	94.9(7)
O(310)-Cd(20)-O(43)	75.7(7)
O(313)-Cd(20)-O(314)	56.2(7)
O(310)-Cd(20)-O(314)	122.3(7)
O(43)-Cd(20)-O(314)	142.9(7)
O(313)-Cd(20)-O(44)	99.8(7)
O(310)-Cd(20)-O(44)	141.8(6)
O(43)-Cd(20)-O(44)	66.9(6)
O(314)-Cd(20)-O(44)	93.4(7)
O(313)-Cd(20)-Cl(7)	141.4(6)
O(310)-Cd(20)-Cl(7)	89.3(6)
O(43)-Cd(20)-Cl(7)	122.3(6)
O(314)-Cd(20)-Cl(7)	92.0(5)
O(44)-Cd(20)-Cl(7)	103.7(5)
O(43)-Cd(21)-O(47)	145.7(7)
O(43)-Cd(21)-N(22)	84.2(8)
O(47)-Cd(21)-N(22)	94.3(8)
O(43)-Cd(21)-O(310)	77.6(7)
O(47)-Cd(21)-O(310)	98.7(7)
N(22)-Cd(21)-O(310)	161.4(8)
O(43)-Cd(21)-O(311)	142.8(7)
O(47)-Cd(21)-O(311)	69.3(6)
N(22)-Cd(21)-O(311)	111.4(7)
O(310)-Cd(21)-O(311)	85.7(7)
O(43)-Cd(21)-O(48)	78.3(7)
O(47)-Cd(21)-O(48)	67.4(6)
N(22)-Cd(21)-O(48)	85.7(8)
O(310)-Cd(21)-O(48)	87.1(7)
O(311)-Cd(21)-O(48)	134.3(6)
O(43)-Cd(21)-O(312)	90.6(7)

O(47)-Cd(21)-O(312)	123.1(6)	O(340)-Cd(23)-O(8)	144.2(7)
N(22)-Cd(21)-O(312)	101.7(7)	O(344)-Cd(23)-O(8)	92.1(7)
O(310)-Cd(21)-O(312)	82.2(7)	O(7)-Cd(23)-O(304)	90.1(7)
O(311)-Cd(21)-O(312)	54.0(6)	O(303)-Cd(23)-O(304)	55.1(7)
O(48)-Cd(21)-O(312)	166.0(6)	O(340)-Cd(23)-O(304)	86.0(7)
O(305)-Cd(22)-N(2)	168.5(8)	O(344)-Cd(23)-O(304)	150.9(7)
O(305)-Cd(22)-O(3)	109.4(7)	O(7)-Cd(24)-N(4)	84.5(10)
N(2)-Cd(22)-O(3)	77.0(8)	O(7)-Cd(24)-O(11)	148.8(7)
O(305)-Cd(22)-O(307)	92.2(7)	N(4)-Cd(24)-O(11)	97.3(9)
N(2)-Cd(22)-O(307)	96.5(8)	O(7)-Cd(24)-O(341)	138.2(7)
O(3)-Cd(22)-O(307)	98.1(7)	N(4)-Cd(24)-O(341)	108.0(10)
O(305)-Cd(22)-O(342)	86.6(7)	O(11)-Cd(24)-O(341)	70.9(7)
N(2)-Cd(22)-O(342)	84.7(8)	O(7)-Cd(24)-O(340)	76.7(7)
O(3)-Cd(22)-O(342)	156.9(7)	N(4)-Cd(24)-O(340)	161.1(10)
O(307)-Cd(22)-O(342)	97.9(7)	O(11)-Cd(24)-O(340)	97.4(7)
O(305)-Cd(22)-O(304)	83.0(7)	O(341)-Cd(24)-O(340)	88.1(7)
N(2)-Cd(22)-O(304)	88.6(8)	O(7)-Cd(24)-O(12)	84.2(8)
O(3)-Cd(22)-O(304)	81.9(7)	N(4)-Cd(24)-O(12)	86.3(9)
O(307)-Cd(22)-O(304)	174.8(7)	O(11)-Cd(24)-O(12)	65.0(7)
O(342)-Cd(22)-O(304)	83.7(7)	O(341)-Cd(24)-O(12)	135.0(7)
O(7)-Cd(23)-O(303)	143.0(7)	O(340)-Cd(24)-O(12)	89.2(7)
O(7)-Cd(23)-O(340)	74.3(7)	O(7)-Cd(24)-O(342)	85.9(7)
O(303)-Cd(23)-O(340)	110.9(8)	N(4)-Cd(24)-O(342)	96.0(8)
O(7)-Cd(23)-O(344)	118.9(7)	O(11)-Cd(24)-O(342)	124.6(6)
O(303)-Cd(23)-O(344)	96.2(7)	O(341)-Cd(24)-O(342)	53.8(7)
O(340)-Cd(23)-O(344)	103.0(7)	O(340)-Cd(24)-O(342)	85.3(6)
O(7)-Cd(23)-O(8)	70.0(7)	O(12)-Cd(24)-O(342)	169.6(7)
O(303)-Cd(23)-O(8)	99.2(7)		

Table S4. Selected Bond Lengths (Å) and Angles (°) for **3**.

Nd(1)-O(15)	2.269(8)	O(19)-Nd(1)-O(4)	144.7(2)
Nd(1)-O(2)	2.280(7)	O(3)-Nd(1)-O(4)	63.5(2)
Nd(1)-O(19)	2.304(7)	O(23)-Nd(1)-O(4)	133.8(2)
Nd(1)-O(3)	2.346(7)	O(21)-Nd(1)-O(4)	109.4(3)
Nd(1)-O(23)	2.350(6)	O(20)-Nd(1)-O(4)	70.7(3)
Nd(1)-O(21)	2.451(9)	O(15)-Nd(1)-N(4)	102.3(3)
Nd(1)-O(20)	2.508(9)	O(2)-Nd(1)-N(4)	171.4(3)
Nd(1)-O(4)	2.626(7)	O(19)-Nd(1)-N(4)	81.8(3)
Nd(1)-N(4)	2.891(10)	O(3)-Nd(1)-N(4)	92.7(3)
Ni(1)-O(9)	2.042(8)	O(23)-Nd(1)-N(4)	99.8(3)
Ni(1)-O(23)	2.112(7)	O(21)-Nd(1)-N(4)	26.1(3)
Ni(1)-N(1)	2.113(9)	O(20)-Nd(1)-N(4)	25.7(3)
Ni(1)-O(5)	2.116(7)	O(4)-Nd(1)-N(4)	89.8(3)
Ni(1)-O(2)	2.141(7)	O(9)-Ni(1)-O(23)	170.7(3)
Ni(1)-N(2)	2.156(8)	O(9)-Ni(1)-N(1)	86.5(3)
Ni(2)-O(10)	2.053(8)	O(23)-Ni(1)-N(1)	100.1(3)
Ni(2)-O(3)	2.066(7)	O(9)-Ni(1)-O(5)	91.8(3)
Ni(2)-O(14)	2.073(7)	O(23)-Ni(1)-O(5)	80.8(3)
Ni(2)-N(3)	2.088(8)	N(1)-Ni(1)-O(5)	172.1(3)
Ni(2)-O(23)	2.120(6)	O(9)-Ni(1)-O(2)	94.4(3)
Ni(2)-O(5)	2.257(7)	O(23)-Ni(1)-O(2)	79.8(3)
O(15)-Nd(1)-O(2)	83.6(3)	N(1)-Ni(1)-O(2)	84.4(3)
O(15)-Nd(1)-O(19)	75.7(3)	O(5)-Ni(1)-O(2)	88.1(3)
O(2)-Nd(1)-O(19)	93.8(3)	O(9)-Ni(1)-N(2)	92.3(3)
O(15)-Nd(1)-O(3)	133.6(3)	O(23)-Ni(1)-N(2)	93.1(3)
O(2)-Nd(1)-O(3)	87.5(3)	N(1)-Ni(1)-N(2)	98.8(3)
O(19)-Nd(1)-O(3)	150.5(2)	O(5)-Ni(1)-N(2)	88.9(3)
O(15)-Nd(1)-O(23)	145.3(2)	O(2)-Ni(1)-N(2)	172.7(3)
O(2)-Nd(1)-O(23)	72.2(2)	O(10)-Ni(2)-O(3)	89.2(3)
O(19)-Nd(1)-O(23)	81.4(2)	O(10)-Ni(2)-O(14)	91.1(3)
O(3)-Nd(1)-O(23)	70.9(2)	O(3)-Ni(2)-O(14)	172.9(3)
O(15)-Nd(1)-O(21)	123.7(3)	O(10)-Ni(2)-N(3)	86.0(3)
O(2)-Nd(1)-O(21)	145.6(3)	O(3)-Ni(2)-N(3)	91.4(3)
O(19)-Nd(1)-O(21)	75.8(3)	O(14)-Ni(2)-N(3)	95.7(3)
O(3)-Nd(1)-O(21)	86.5(3)	O(10)-Ni(2)-O(23)	93.5(3)
O(23)-Nd(1)-O(21)	73.8(2)	O(3)-Ni(2)-O(23)	81.2(3)
O(15)-Nd(1)-O(20)	80.2(3)	O(14)-Ni(2)-O(23)	91.7(3)
O(2)-Nd(1)-O(20)	162.4(3)	N(3)-Ni(2)-O(23)	172.6(3)
O(19)-Nd(1)-O(20)	88.8(3)	O(10)-Ni(2)-O(5)	171.0(3)
O(3)-Nd(1)-O(20)	98.8(3)	O(3)-Ni(2)-O(5)	89.7(3)
O(23)-Nd(1)-O(20)	125.4(3)	O(14)-Ni(2)-O(5)	89.0(3)
O(21)-Nd(1)-O(20)	51.8(3)	N(3)-Ni(2)-O(5)	102.9(3)
O(15)-Nd(1)-O(4)	72.8(2)	O(23)-Ni(2)-O(5)	77.5(2)
O(2)-Nd(1)-O(4)	98.0(3)		
